# Loss of vacuolar-type H^+^-ATPase induces caspase-independent necrosis-like death of hair cells in zebrafish neuromasts

**DOI:** 10.1242/dmm.048997

**Published:** 2021-07-23

**Authors:** Peu Santra, Jeffrey D. Amack

**Affiliations:** 1 Department of Cell and Developmental Biology, State University of New York Upstate Medical University, Syracuse, NY 13210, USA; 2 BioInspired Syracuse: Institute for Material and Living Systems, Syracuse, NY 13244, USA

**Keywords:** Vacuolar-type H^+^-ATPase (V-ATPase), Zebrafish, Neuromast hair cell, Necrosis-like cell death, Mitochondrial membrane potential

## Abstract

The vacuolar-type H^+^-ATPase (V-ATPase) is a multi-subunit proton pump that regulates cellular pH. V-ATPase activity modulates several cellular processes, but cell-type-specific functions remain poorly understood. Patients with mutations in specific V-ATPase subunits can develop sensorineural deafness, but the underlying mechanisms are unclear. Here, we show that V-ATPase mutations disrupt the formation of zebrafish neuromasts, which serve as a model to investigate hearing loss. V-ATPase mutant neuromasts are small and contain pyknotic nuclei that denote dying cells. Molecular markers and live imaging show that loss of V-ATPase induces mechanosensory hair cells in neuromasts, but not neighboring support cells, to undergo caspase-independent necrosis-like cell death. This is the first demonstration that loss of V-ATPase can lead to necrosis-like cell death in a specific cell type *in vivo.* Mechanistically, loss of V-ATPase reduces mitochondrial membrane potential in hair cells. Modulating the mitochondrial permeability transition pore, which regulates mitochondrial membrane potential, improves hair cell survival. These results have implications for understanding the causes of sensorineural deafness, and more broadly, reveal functions for V-ATPase in promoting survival of a specific cell type *in vivo*.

## Abstract (precis)

**Summary:** Vacuolar-type H^+^-ATPase (V-ATPase) mutations cause necrosis-like death of mechanosensory hair cells in zebrafish. This work identifies new cell-type-specific V-ATPase functions and may help understand the causes of sensorineural deafness.

## INTRODUCTION

**Fig. 1.**
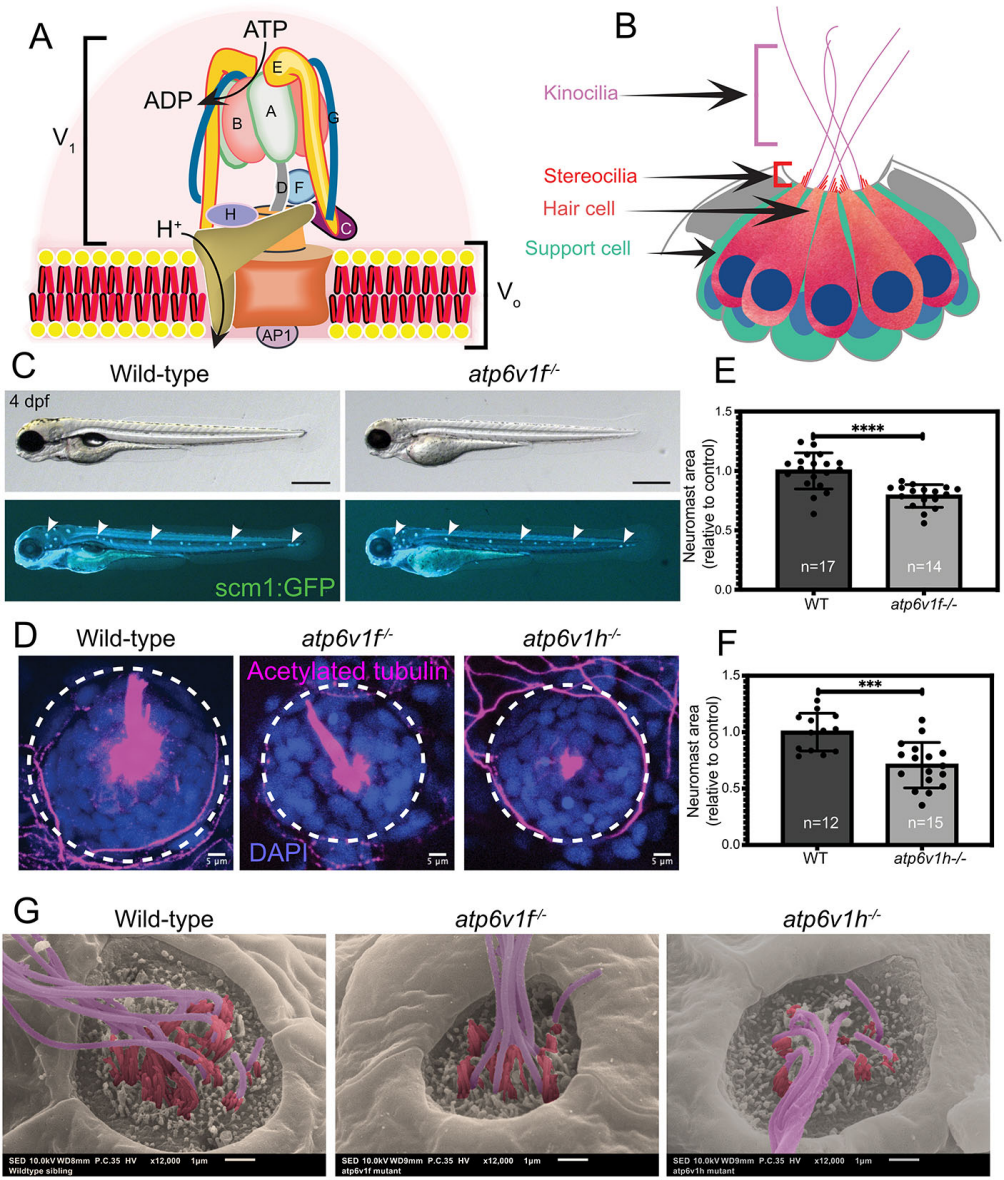
**V-ATPase mutant embryos show neuromast defects.** (A) Diagram of the V-ATPase holoenzyme complex. The cytosolic V_1_ domain contains subunits A-H and hydrolyzes ATP, and the V_o_ domain inserts into membrane and translocates protons (H^+^). The accessory protein AP1 interacts with the V_o_ domain. Adapted from Collins and Forgac (2018) and Abbas et al. (2020) under the terms of the CC-BY 4.0 license. (B) Diagram of a lateral view of a zebrafish neuromast with centrally organized hair cells (orange) surrounded by support cells (green). Each hair cell has a single kinocilium (pink), a staircase of stereocilia (red) and a nucleus (blue). (C) Wild-type and *atp6v1f^−/−^* embryos at 4 dpf expressing the *Tg(scm1:GFP)* transgene that labels lateral line neuromasts (white arrowheads). Scale bars: 500 µm. (D) Top-down view of neuromasts in wild-type, *atp6v1f^−/−^* and *atp6v1h^−/−^* embryos labeled with acetylated tubulin to mark hair cells (magenta) and DAPI to stain nuclei (blue). Dashed line circles indicate the approximate neuromast boundary. (E,F) Quantification of neuromast area in *atp6v1f^−/−^* (E) and *atp6v1h^−/−^* (F) embryos at 4 dpf relative to wild-type (WT) siblings. *n*=number of embryos examined. ***P=0.0002, ****P<0.0001 by unpaired Student's *t*-test with Welch's correction. (G) Pseudo-colored scanning electron micrographs of wild-type, *atp6v1f^−/−^* and *atp6v1h^−/−^* neuromasts at 4 dpf with pink representing kinocilia and red representing stereocilia. Scale bars: 1 µm.

The vacuolar-type H^+^-ATPase (V-ATPase) protein complex localizes to membranes of organelles and vesicles and translocates protons (H^+^) into their lumens by hydrolyzing ATP (Nishi and Forgac, 2002; Marshansky and Futai, 2008; Holliday, 2014). Tightly regulated V-ATPase activity maintains proper pH in these compartments, which is critical for multiple cellular functions that include vesicle trafficking, protein degradation and ion homeostasis (Kane, 2007; Vasanthakumar and Rubinstein, 2020). In some cell types, V-ATPase also localizes to the plasma membrane to regulate extracellular pH. Inhibition of V-ATPase activity can impact cell proliferation, migration or survival (Cotter et al., 2015). The V-ATPase holoenzyme is composed of a transmembrane V_o_ domain and a cytosolic V_1_ domain, and each of these domains is comprised of multiple core subunits (Marshansky et al., 2014; Cotter et al., 2015) (Fig. 1A). Some of these subunits have multiple isoforms, which can show tissue-specific expression and/or function. There are accessory proteins that associate with the V-ATPase in some contexts. Although complete loss of V-ATPase function is embryonic lethal in animal models (Davies et al., 1996; Inoue et al., 1999; Sun-Wada et al., 2000), recessive loss-of-function mutations in specific human subunits can cause disorders that affect distinct tissue types, which include distal renal tubular acidosis (kidney) (Karet et al., 1999; Stover et al., 2002), osteopetrosis (bone) (Kornak et al., 2000) and cutis laxa (skin) (Kornak et al., 2008). These findings reveal that V-ATPase has cell-type-specific functions in different organs, but these functions are only beginning to be understood.

The zebrafish embryo provides a useful system to investigate *in vivo* V-ATPase functions because embryos develop externally and V-ATPase subunits are maternally supplied (Adams et al., 2006; Nuckels et al., 2009; Gokey et al., 2015). This allows analysis of cellular processes in zygotic V-ATPase mutants, as well as acute loss-of-function studies using gene knockdown or pharmacological approaches. In previous work, we used zebrafish to analyze V-ATPase functions in cells that give rise to the left-right organizer, an embryonic structure that establishes the left-right body axis in vertebrate embryos (Gokey et al., 2015). We found that small-molecule inhibition of V-ATPase activity, or gene knockdown of the *atp6ap1b* gene that encodes a V-ATPase accessory protein, reduced the size of the left-right organizer and caused organ laterality defects. Compromised V-ATPase activity was found to reduce proliferation of cells that give rise to the left-right organizer. Analysis of a loss-of-function mutation in *atp6ap1b* also revealed defects in neuromasts along the zebrafish lateral line. Neuromast size was reduced in *atp6ap1b* mutants, but the mechanisms by which V-ATPase impacts neuromast development were not pursued (Gokey et al., 2015).

Here, we focused on the zebrafish neuromast to further investigate cell-type-specific V-ATPase functions. Neuromasts are sensory organs in the lateral line system on the surface of aquatic vertebrates that detect directional water movement (Lush and Piotrowski, 2014; Pickett and Raible, 2019). Each neuromast is comprised of non-sensory support cells that surround a central cluster of mechanosensory hair cells (Fig. 1B). Hair cells extend a single long microtubule-based kinocilium and a staircase-like bundle of several shorter actin-based stereocilia from its apical surface. Mechanical bending of stereocilia opens cation channels to generate electrical impulses that are carried by neurons to the brain (Gillespie and Müller, 2009). Hair cells in the zebrafish neuromast are structurally and functionally similar to hair cells in other vertebrates (Whitfield, 2002; Nicolson, 2005). Hair cells in human inner ear convert sound vibrations into electrical signals to provide the basis for hearing. Some patients with distal renal tubular acidosis that have mutations in *ATP6V1B1* or *ATP6V0A4*, which encode the V-ATPase V_1_B1 and V_o_a4 subunits, respectively, develop sensorineural hearing loss, which is typically caused by damage to the sensory hair cells and/or nerve fibers of the inner ear (Karet et al., 1999; Stover et al., 2002; Vargas-Poussou et al., 2006; Subasioglu Uzak et al., 2013). How these mutations impact hearing is not completely understood. *Atp6v1b1* and *Atp6v0a4* knockout mice can show severe hearing loss and enlarged endolymphatic compartments in the inner ear, but hair cells appear normal (Norgett et al., 2012; Lorente-Cánovas et al., 2013; Tian et al., 2017). However, *Atp6v1b1* null mutations in a different strain of mice have no effect on hearing (Dou et al., 2003), indicating that genetic background has a significant impact. Because these mutations affect subunits with multiple isoforms, there may be redundancy and/or compensation mechanisms to support V-ATPase functions in the inner ear. In zebrafish, a mutation in *rabconnectin 3a* (also known as *dmxl2*), which encodes a V-ATPase assembly factor, alters acidification of synaptic vesicles and reduces synaptic transmission from neuromast hair cells (Einhorn et al., 2012), which suggests specific functions for V-ATPase in hair cells. However, loss of *rabconnectin 3a* only alters the pH regulation of synaptic vesicles. Thus, in both mouse and zebrafish models, the impact of complete loss of V-ATPase function on hair cells remains unknown.

Using mutations in genes encoding core V-ATPase subunits – *atp6v1f* or *atp6v1h* – we show that loss of V-ATPase activity alters zebrafish neuromast formation. We find that defects in neuromast size and architecture in V-ATPase mutants are due to reduced survival of hair cells. Additional analyses indicate that mutant hair cells, but not surrounding support cells, undergo necrosis-like cell death that is independent of caspase activity. As loss of V-ATPase has typically been associated with apoptotic cell death, this is the first example of necrosis-like cell death *in vivo*. At the molecular level, loss of V-ATPase activity results in depolarization of the mitochondrial membrane in hair cells, which has previously been linked to mitochondrial dysfunction and, ultimately, cell death. Directly inhibiting opening of the mitochondrial permeability transition pore (mPTP), which regulates mitochondrial membrane polarization, reduced hair cell death. In addition, blocking the mitochondrial calcium uniporter (MCU) that controls calcium ion (Ca^2+^) influx into mitochondria, which also regulates mPTP opening, improved hair cell survival. Taken together, these results uncover novel *in vivo* cell-type-specific functions for V-ATPase that regulate mitochondrial health in hair cells, which contributes to cell survival. This protective function for V-ATPase in hair cells may contribute to our understanding of causes of sensorineural deafness.

## RESULTS

### Mutations in core V-ATPase subunits cause neuromast defects

Previous work in zebrafish revealed that loss of the V-ATPase accessory protein Atp6ap1b reduced neuromast size in the lateral line (Gokey et al., 2015). Recent work indicates that ATP6AP1 in humans (Wang et al., 2020) and its homolog Voa1 in yeast (Jansen et al., 2016) are central subunits involved in V-ATPase assembly. However, in zebrafish, two genes – *atp6ap1a* and *atp6ap1b* – encode ATP6AP1-like proteins, and the function(s) of each of these subunits remain unknown. To further investigate the function of V-ATPase activity in neuromasts, we chose to analyze two previously described loss-of-function mutations that disrupt expression of the V1F (*atp6v1f^hi1988Tg^* allele) or V1H (*atp6v1h^hi923Tg^* allele) subunits (Nuckels et al., 2009), because each is an essential subunit encoded by a single gene in zebrafish. Work in yeast has demonstrated that both V_1_F and V_1_H are required for V-ATPase activity (Ho et al., 1993; Nelson et al., 1994). Zebrafish homozygous zygotic *atp6v1f^hi1988Tg^* and *atp6v1h^hi923Tg^* mutants (referred to here as *atp6v1f^−/−^* and *atp6v1h^−/−^*) are indistinguishable from wild-type siblings during early development but become easily identifiable at 2 days post-fertilization (dpf) due to hypopigmentation (Nuckels et al., 2009) (Fig. 1C; Fig. S1A,B). Zygotic *atp6v1f^−/−^* and *atp6v1h^−/−^* mutants continue to develop for several days, likely supported by maternal supply of subunit protein, but both mutations are ultimately lethal, with mutant larvae dying after 5 dpf. To analyze lateral line neuromast development in V-ATPase mutants, we used the *Tg(scm1:GFP)* transgene that expresses GFP in support cells (Behra et al., 2009) and the *Tg(cldnb:lynEGFP)* transgene that expresses EGFP in all neuromast cells (Haas and Gilmour, 2006) as markers. The number and pattern of neuromasts in the head [anterior lateral line (aLL)] and trunk (posterior lateral line) at 4 dpf was similar among V-ATPase mutants and wild-type siblings (Fig. 1C; Fig. S1C). To analyze individual neuromasts, we focused on the caudal-cranial region of the aLL, specifically the otic O1, O2 and middle MI1 neuromasts (Van Trump and McHenry, 2008), and used acetylated tubulin antibodies to mark hair cells and 4′,6-diamidino-2-phenylindole (DAPI) to identify nuclei. At 4 dpf, neuromast area was smaller in *atp6v1f^−/−^* and *atp6v1h^−/−^* mutants, compared to wild-type siblings (Fig. 1D-F). To more closely examine neuromast architecture, we performed scanning electron microscopy (SEM) on *atp6v1f^−/^*^−^, *atp6v1h^−/−^* and wild-type embryos at 4 dpf. V-ATPase mutant neuromasts appeared smaller and malformed compared to wild-type neuromasts (Fig. 1G). SEM revealed that both kinocilia and stereocilia were present on V-ATPase mutant hair cells, but the number of these structures appeared to be reduced (Fig. 1G). These results indicate that loss of an essential V-ATPase subunit results in neuromast phenotypes, implicating V-ATPase activity in neuromast development.

### V-ATPase subunit expression in *atp6v1f^−/^*^−^ and *atp6v1h^−/−^* mutants

**Fig. 2.**
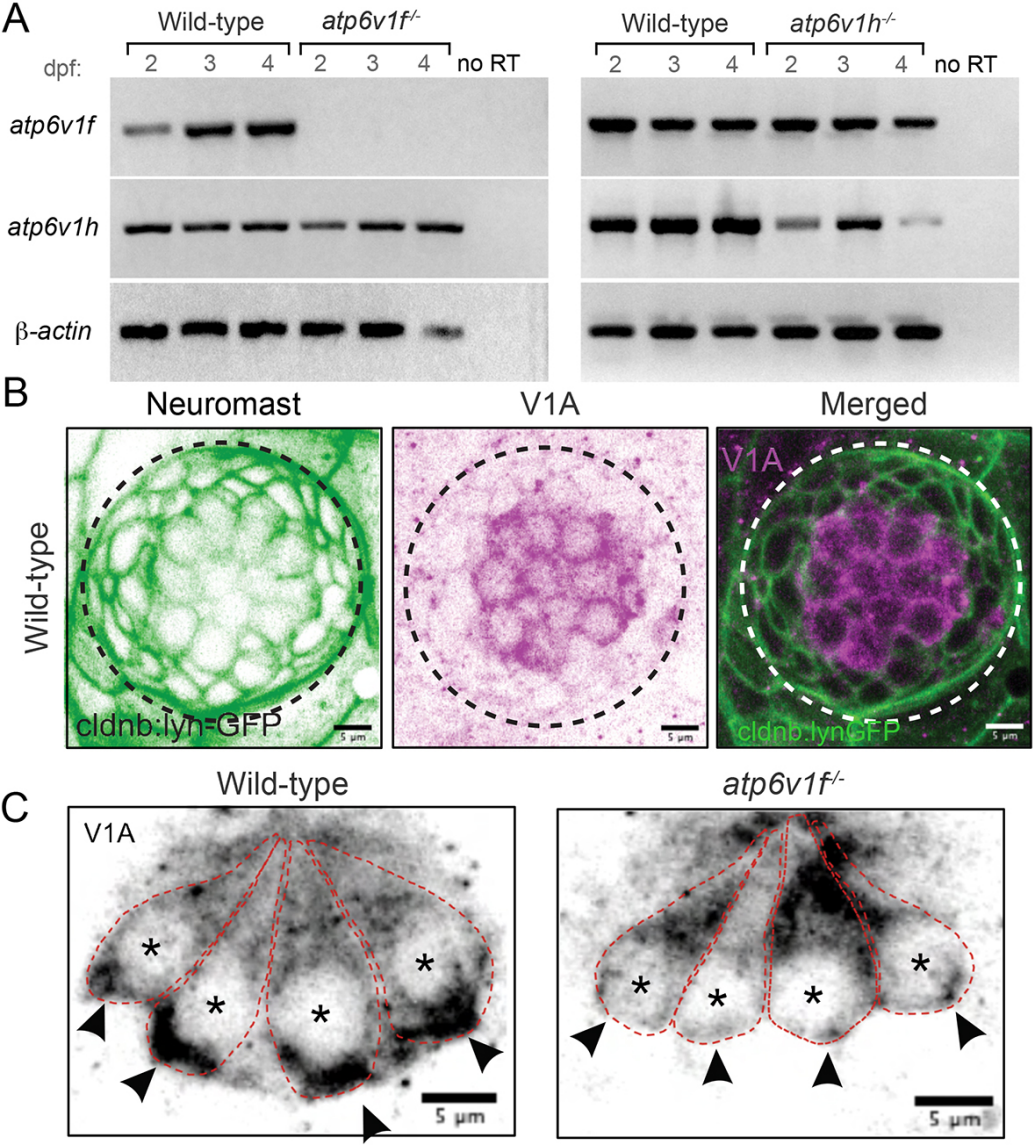
**Expression of V-ATPase subunits in wild-type and V-ATPase mutant zebrafish.** (A) Reverse transcriptase (RT)-PCR analysis of total mRNA from wild-type and V-ATPase mutant embryos at 2, 3 and 4 dpf. *atp6v1f* mRNA is not detected in *atp6v1f^−/−^* embryos, whereas *atp6v1h* mRNA is detected in the *atp6v1h^−/−^* embryos. β-actin mRNA was amplified as a positive control, and reactions without reverse transcriptase (no RT) were negative controls. (B) Antibodies against the V-ATPase V_1_A subunit (magenta) show enriched staining in centrally localized hair cells in a wild-type neuromast labeled by *Tg(cldnb:lynEGFP)* expression (green). Dashed line circles indicate the neuromast boundary. (C) Optical sections of hair cells reveal that the V_1_A subunit localizes throughout wild-type hair cells with an accumulation in the basal region. This basal localization is disrupted in *atp6v1f^−/−^* mutant hair cells. Approximate boundaries of individual hair cells are outlined, and asterisks mark hair cell nuclei. Arrowheads indicate basal accumulation of V_1_A in wild-type hair cells, and lack thereof in *atp6v1f^−/−^* hair cells.

The *atp6v1f^hi1988Tg^* and *atp6v1h^hi923Tg^* mutant alleles, identified in a large-scale insertion mutagenesis screen (Amsterdam et al., 2004), have a retroviral insertion in the first exon (*atp6v1f*) or first intron (*at6v1h*) that cause a reduction in zygotic transcription of *atpv1f* or *atp6v1h*, respectively, when analyzed at 5 dpf (Nuckels et al., 2009). To assess expression at earlier time points in *atp6v1f^−/^*^−^ and *atp6v1h^−/−^* mutants, we collected total RNA from embryos at 2, 3 and 4 dpf for non-quantitative reverse transcriptase (RT)-PCR. Expression of *atp6v1f* mRNA was undetected at each of the time points in *atp6v1f^−/^*^−^ mutants, whereas other representative V-ATPase subunit transcripts were detected (Fig. 2A; Fig. S2). In *atp6v1h^−/^*^−^ mutants, however, *atp6v1h* mRNA was detected at all three time points (Fig. 2A). Similar to *atp6v1f^−/^*^−^ mutants, other subunit mRNAs were detected in *atp6v1h^−/^*^−^ mutants (Fig. 2A; Fig. S2). Based on these results, we focused primarily on using *atp6v1f^−/^*^−^ mutants for subsequent experiments.

To determine the impact of *atp6v1f^−/^*^−^ mutation on V-ATPase subunit protein expression in neuromasts, we used a previously described antibody against the V_1_A subunit (Atp6v1a) (Einhorn et al., 2012; Gokey et al., 2015) in whole-embryo immunofluorescence experiments. Interestingly, we found V-ATPase staining enriched in the hair cells relative to the surrounding support cells in wild-type neuromasts marked by *Tg(cldnb:lynEGFP)* transgene (Fig. 2B). Using confocal optical sections to focus on hair cells, we detected V_1_A staining throughout wild-type cells, with a notable accumulation in the basal region (Fig. 2C), which has been previously reported (Einhorn et al., 2012). In *atp6v1f^−/−^* mutants, the basal accumulation of V_1_A protein was reduced in hair cells (Fig. 2C). These results suggest that loss of Atp6v1f alters the localization and/or assembly of the V-ATPase holoenzyme in hair cells, which would be predicted to compromise V-ATPase function.

### Defects in organelle acidification and autophagy indicate that V-ATPase activity is compromised in *atp6v1f**^−/^***^−^ mutant hair cells

**Fig. 3.**
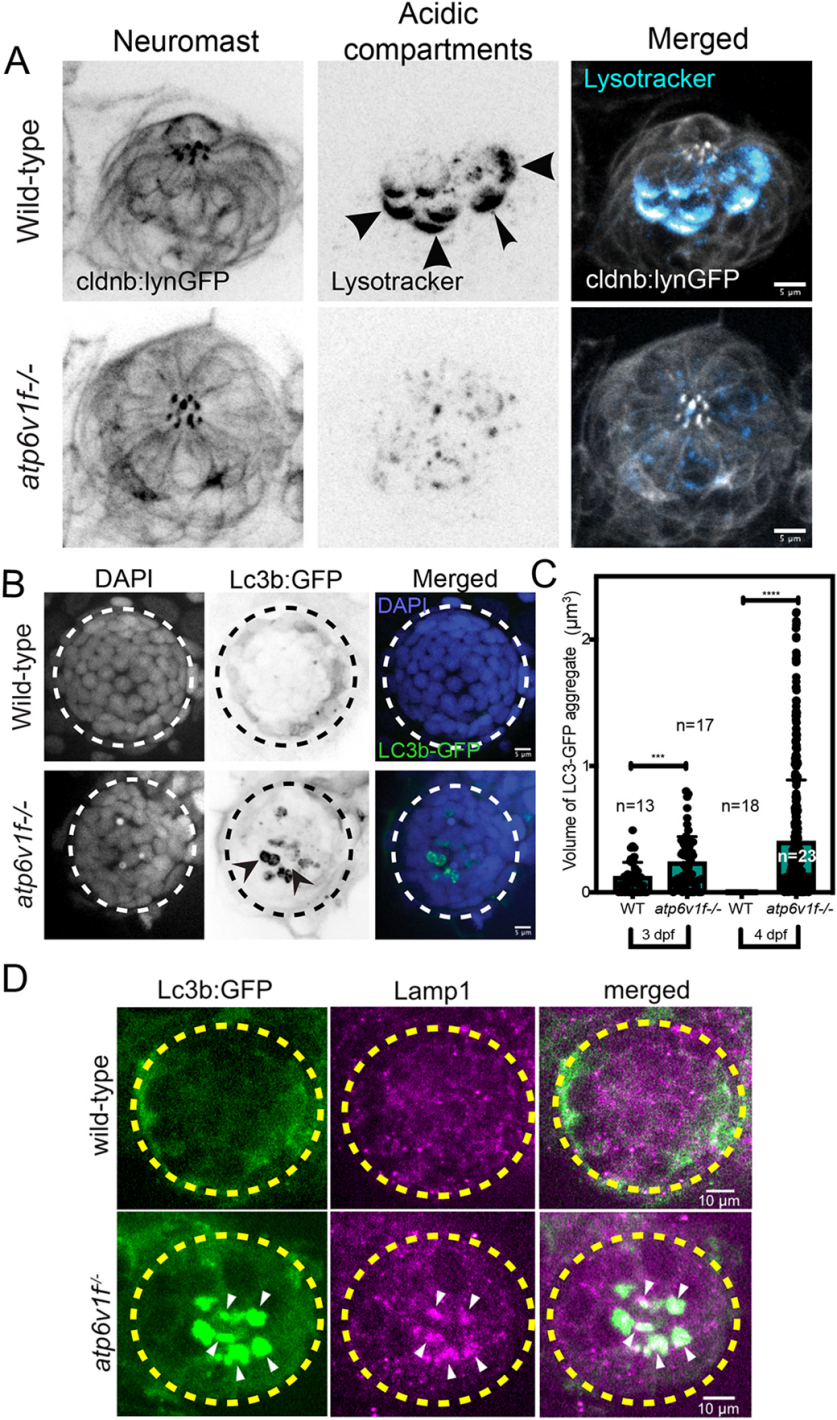
**V-ATPase loss of function alters pH and induces autophagy defects in neuromasts.** (A) Lysotracker staining in a wild-type neuromast at 4 dpf was reduced in *atp6v1f^−/−^* mutants. Neuromast cells are marked by *Tg(cldnb:lynEGFP)* transgene expression. Arrowheads point out basal accumulation of Lysotracker in wild-type hair cells. Scale bars: 5 µm. (B) Wild-type and *atp6v1f^−/−^* mutant neuromasts at 4 dpf stained for Lc3b-GFP (green) and nuclei using DAPI (blue). Arrowheads point out Lc3b-GFP aggregates. White and black dashed line circles indicate the neuromast boundary. (C) Quantification of volume of individual Lc3b-GFP aggregates in neuromasts from wild-type and *atp6v1f^−/−^* embryos. The number of data points reflects the finding that each embryo can have multiple neuromast aggregates. *n*=number of embryos. ***P=0.0005 and ****P<0.0001 by unpaired Student's *t*-test with Welch's correction. (D) Anti-Lamp1 antibody staining shows colocalization with Lc3b-GFP aggregates in *atp6v1f^−/−^* mutant neuromast. Yellow dashed line circles indicate hair cell cluster within a neuromast.

V-ATPase activity is critical for acidifying the lumens of vesicles and organelles. Therefore, to directly assess V-ATPase activity in hair cells we used the vital dye Lysotracker that labels lysosomes and other acidic cellular compartments. In wild-type neuromasts marked by *Tg(cldnb:lynEGFP)* transgene expression, we observed intense Lysotracker staining in the basal region of hair cells (Fig. 3A), which is reminiscent of previously reported V_1_A subunit accumulation (Fig. 2C) (Einhorn et al., 2012). Also, similar to V_1_A immunostaining, Lysotracker intensity was higher in hair cells than in surrounding support cells. In contrast to wild-type hair cells, Lysotracker staining was significantly reduced in *atp6v1f^−/^*^−^ mutant hair cells at 4 dpf (Fig. 3A), indicating a defect in acidification.

As a functional test for loss of V-ATPase activity in *atp6v1f^−/^*^−^ mutants, we assessed autophagy. Autophagy – the process of degrading, recycling and reusing cellular components – depends on fusion of autophagosomes with acidified and functional lysosomes that contain hydrolyzing enzymes (Parzych and Klionsky, 2014; Saha et al., 2018). In several cellular contexts, V-ATPase mutations result in an accumulation of autophagosomes, which denotes a block in autophagic flux (Nakamura et al., 1997; Mangieri et al., 2014; Mauvezin et al., 2015; Xia et al., 2019). Because hair cells are known to upregulate autophagy as a survival mechanism in response to stress (He et al., 2017), we analyzed autophagy in *atp6v1f^−/^*^−^ mutant hair cells. To assess autophagic flux, we used transgenic *Tg(CMV:EGFP-map1lc3b)* embryos that express GFP fused with the LC3b protein (LC3b-GFP) marking autophagosomes in living embryos (He et al., 2009). At 3 dpf and 4 dpf, *atp6v1f^−/^*^−^ mutant neuromasts show an accumulation of LC3b-GFP aggregates that are not present in wild-type neuromasts (Fig. 3B,C). Live imaging of LC3b-GFP in *Tg(myo6b:tdtomato)* embryos, in which hair cells express red fluorescent tdTomato specifically in hair cells (Toro et al., 2015), revealed that LC3b-GFP aggregates were present in hair cells and not in surrounding support cells (Fig. S3). Next, immunostaining with the lysosomal marker Lamp1 revealed Lamp1-positive puncta localized with LC3b-GFP aggregates in V-ATPase mutant hair cells (Fig. 3D). These results suggest that autophagosomes can fuse with lysosomes in V-ATPase mutant hair cells, and that the aggregation of autophagosomes is due to defective lysosomes that are unable to degrade the contents. Taken together, these results indicate that V-ATPase activity is compromised in *atp6v1f^−/^*^−^ mutant neuromasts.

### The number of hair cells is reduced in V-ATPase mutant neuromasts

**Fig. 4.**
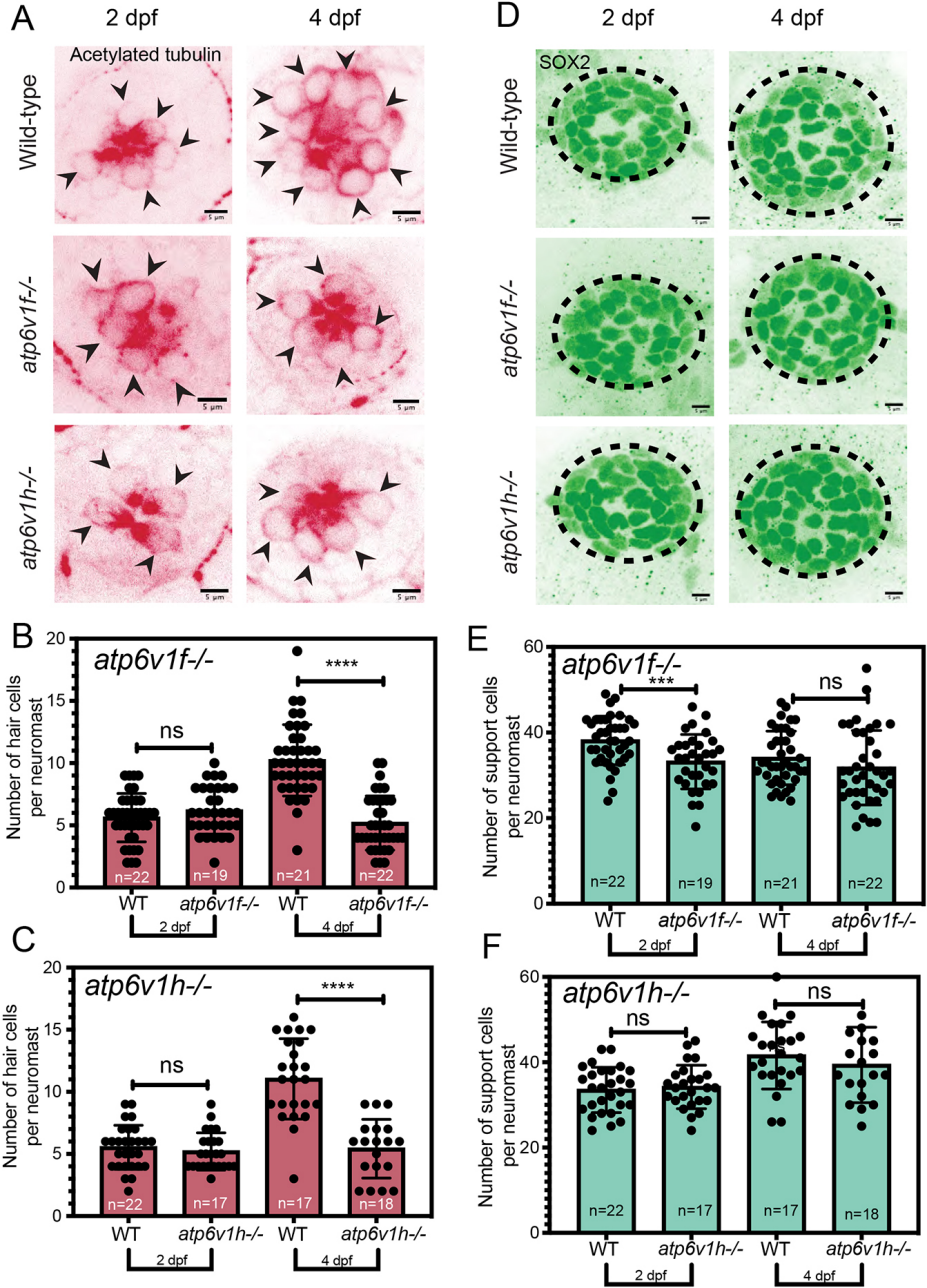
**V-ATPase loss reduces the number of hair cells in neuromasts.** (A) Acetylated tubulin staining detects hair cells in wild-type, *atp6v1f^−/−^* and *atp6v1h^−/−^* neuromasts at 2 dpf and 4 dpf. Arrowheads point to individual hair cells. (B,C) The number of hair cells per neuromast at 2 dpf and 4 dpf in *atp6v1f^−/−^* embryos and wild-type siblings (B), and *atp6v1h^−/−^* and wild-type siblings (C). (D) Sox2 staining labels neuromast support cells at 2 dpf and 4 dpf. Dashed line circles indicate the neuromast boundary. (E,F) The number of support cells per neuromast at 2 dpf and 4 dpf in *atp6v1f^−/−^* embryos and wild-type siblings (E), and *atp6v1h^−/−^* and wild-type siblings (F). *n*=number of embryos. ***P=0.0009 and ****P<0.0001 by unpaired Student's *t*-test with Welch's correction. ns, not significant.

To understand why neuromasts are smaller in V-ATPase mutants, we used molecular markers to determine which neuromast cell type(s) are affected by loss of V-ATPase activity. In immunostaining experiments, we used acetylated tubulin as a marker to count the number of hair cells (Harris et al., 2003; Sarrazin et al., 2006) in aLL neuromasts. At 2 dpf, *atp6v1f^−/−^*, *atp6v1h^−/−^* mutants and wild type have similar hair cell numbers (Fig. 4A-C). However, at 4 dpf, the number of hair cells was significantly reduced in *atp6v1f^−/−^* and *atp6v1h^−/−^* mutant neuromasts (Fig. 4A-C). We next used antibodies against Parvalbumin as a marker of mature hair cells (Lopez-Schier and Hudspeth, 2005). Similar to acetylated tubulin staining results, the number of Parvalbumin-positive hair cells at 4 dpf was reduced in mutants compared to wild-type siblings (Fig. S4). The reduction in hair cells in V-ATPase mutant neuromasts is consistent with the reduction in apical hair cell structures – kinocilia and stereocilia – observed using SEM (Fig. 1G). Using anti-Sox2 antibodies to label neuromast support cells (Hernández et al., 2007; Froehlicher et al., 2009; Montalbano et al., 2018) revealed largely similar numbers of support cells between wild type and V-ATPase mutants (Fig. 4D). We detected a statistically significant difference in support cell number at 2 dpf in *atp6v1f^−/−^* mutants, but there was no difference at 4 dpf when neuromast size is reduced (Fig. 4E). In *atp6v1h^−/−^* mutants, there was no difference in the number of support cells at 2 dpf or 4 dpf (Fig. 4F). Together, these findings indicate that the smaller neuromast size at 4 dpf in V-ATPase mutants is due to a reduced number of hair cells.

### V-ATPase mutant hair cells undergo Caspase 3-independent necrosis-like cell death

**Fig. 5.**
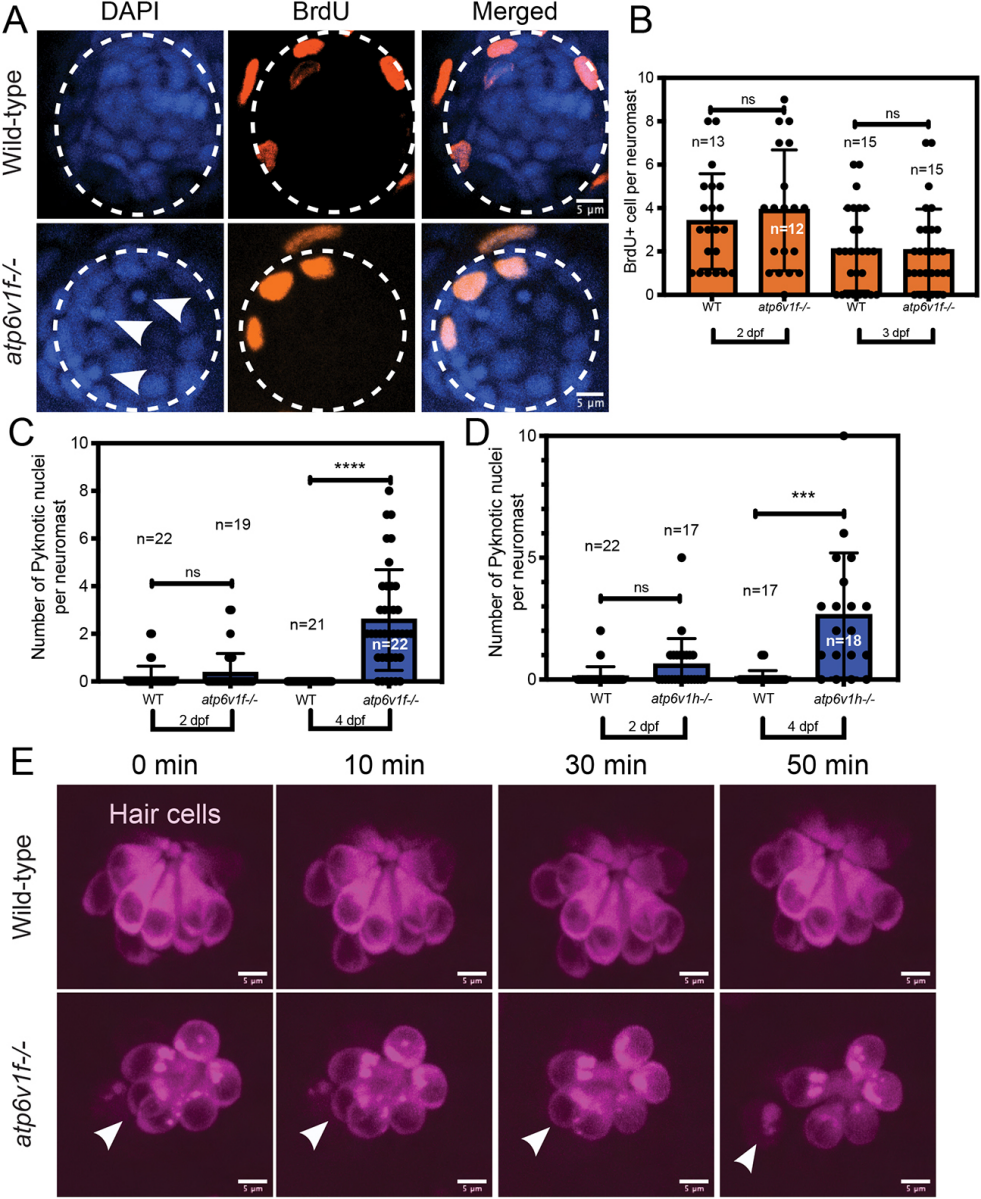
**Loss of V-ATPase does not alter proliferation but induces hair cell death in mutant neuromasts.** (A,B) A 1-h pulse with BrdU results in a similar number of BrdU-positive cells in wild-type and *atp6v1f^−/−^* neuromasts at 2 dpf and 3 dpf. Representative images show BrdU-labeled cells (orange) and DAPI-stained neuromast nuclei (blue). Arrowheads denote pyknotic nuclei. Dashed line circles indicate the neuromast boundary. (B) Quantification of the number of BrdU-labeled cells per neuromast at 2 dpf and 3 dpf. (C,D) The number of pyknotic nuclei increases from 2 dpf through 4 dpf in both *atp6v1f^−/−^* (C) and *atp6v1h^−/−^* (D) neuromasts. (E) Snapshots from live time-lapse imaging of wild-type and *atp6v1f^−/−^* hair cells marked by *Tg(myo6b:tdtomato)* expression. The arrowhead follows a single hair cell in the *atp6v1f^−/−^* neuromast over time as it dies. Fluorescent tdTomato protein accumulates into aggregates that are reminiscent of LC3b-GFP aggregates (see Fig. 3). Wild-type hair cells remained healthy. Scale bars: 5 µm. *n*=number of embryos. ***P=0.0009 and ****P<0.0001 by unpaired Student's *t*-test with Welch's correction. ns, not significant.

We next wanted to understand the cellular mechanism(s) that underlie the reduced number of hair cells in V-ATPase mutant neuromasts. Fewer hair cells could be due to defects in cell proliferation and/or cell survival. We first used bromodeoxyuridine (BrdU) incorporation or phospho-Histone H3 (pHH3) immunostaining to detect proliferating cells in neuromasts. Between 2 dpf and 4 dpf, cell proliferation rates are low in neuromasts, and we did not detect differences between wild type and *atp6v1f^−/−^* mutants (Fig. 5A,B; Fig. S5). However, while investigating cell division, DAPI staining of DNA revealed the presence of pyknotic nuclei in V-ATPase mutant neuromasts (arrowheads in Fig. 5A). Pyknosis, the irreversible condensation of chromatin during cell death, can be a result of either apoptotic or necrotic cell death (Hou et al., 2016). There was a significant increase in the number of pyknotic nuclei between 2 dpf and 4 dpf in *atp6v1f^−/−^* neuromasts, whereas wild-type siblings had few or no pyknotic nuclei (Fig. 5C). A similar increase in pyknotic nuclei was observed in *atp6v1h^−/−^* neuromasts (Fig. 5D). We next used the fluorescent vital dye Ethidium Homodimer III (EthD III) as a cell death marker in live imaging experiments. In mammalian cell cultures, EthD III is impermeant to living cells but binds DNA in necrotic cells and late apoptotic cells that have lost membrane integrity. Interestingly, we found that EthD III accumulates in the cytoplasm of what appear to be healthy, intact hair cells in wild-type neuromasts at 4 dpf (Fig. S6A). EthD III was also found to colocalize with Hoechst staining of DNA in pyknotic nuclei in *atp6v1f^−/−^* neuromasts (Fig. S6B). Fortuitously, Hoechst stained the nuclei in hair cells. In positive-control experiments, EthD III labeled pyknotic nuclei in wild-type neuromasts treated with the aminoglycoside antibiotic neomycin, which is known to induce hair cell death (Fig. S6C) (Owens et al., 2007). These results suggested that loss of V-ATPase does not alter support cell proliferation but reduces cell survival in neuromasts.

The increase in the number of pyknotic nuclei between 2 dpf and 4 dpf (Fig. 5C,D) coincides with the reduction in hair cells in V-ATPase mutants (Fig. 4B,C), which suggests that hair cells are dying in mutant neuromasts. To directly visualize the cell type(s) dying, we followed neuromast development in live transgenic embryos with fluorescently labeled support cells [marked by *Tg(scm1:GFP)* expression] and hair cells [marked by *Tg(myo6b:tdtomato)* expression]. Live imaging captured the swelling and bursting of hair cells in *atp6v1f^−/−^* mutant neuromasts (Fig. 5E), whereas support cells remained healthy over time (Movies 1 and 2). No dying cells were observed in wild-type siblings imaged under the same conditions (Fig. 5E; Movies 3 and 4). The specific death of hair cells, and not neighboring support cells, identifies hair cells as being highly dependent on V-ATPase activity for survival. Taken together, these results indicate that hair cell death is the cellular mechanism that leads to fewer hair cells and smaller neuromast size in V-ATPase mutants.

**Fig. 6.**
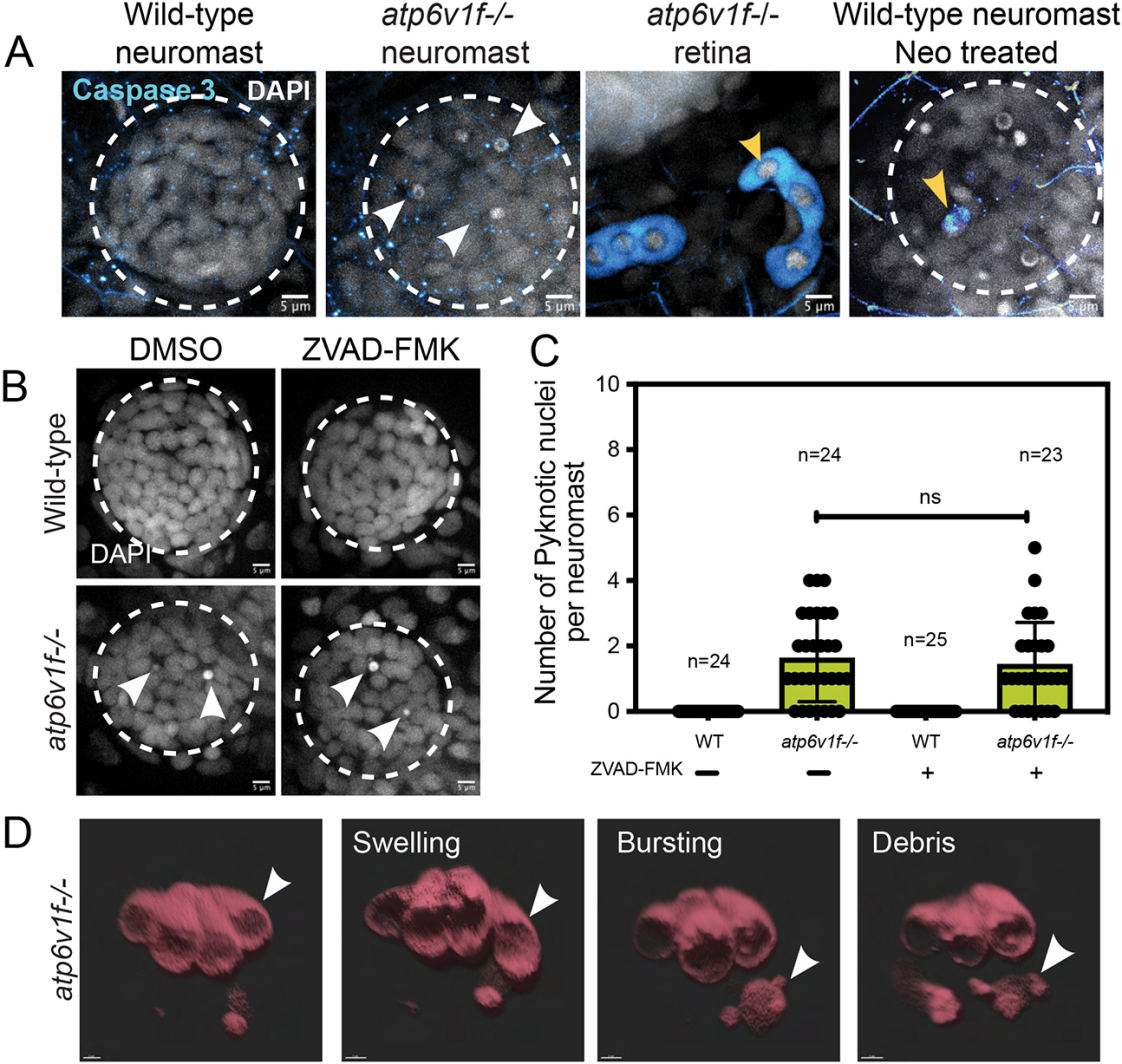
**V-ATPase mutant hair cell death is independent of Caspase 3 and morphologically resembles necrosis.** (A) Cleaved Caspase 3 staining was absent in wild-type and *atp6v1f^−/−^* mutant neuromasts at 4 dpf, but was detected in the retina of *atp6v1f^−/−^* embryos, and in wild-type hair cells treated with neomycin (Neo) that induces Caspase 3 activation (yellow arrowheads). Nuclei were detected using DAPI. White arrowheads indicate pyknotic nuclei. (B) DAPI staining of neuromast nuclei at 4 dpf in wild-type and *atp6v1f^−/−^* embryos treated with either DMSO (control) or 300 µM ZVAD-FMK from 2 dpf to 4 dpf. White arrowheads indicate pyknotic nuclei. Dashed line circles indicate the neuromast boundary. Scale bars: 5 µm. (C) Quantification of pyknotic nuclei per neuromast in control and 300 µM ZVAD-FMK-treated embryos. *n*=number of embryos. ns, not significant by unpaired Student's *t*-test with Welch's correction. (D) 3D rendering of time-lapse snapshots of *Tg(myo6b:tdtomato); atp6v1f^−/−^* hair cells undergoing necrosis-like morphological changes. The white arrowhead follows one hair cell swelling and then bursting over time. Scale bars: 3 µm.

Next, we sought to identify the mode of cell death of V-ATPase mutant hair cells. Hair cells facing cellular stresses, such as the presence of neomycin or other aminoglycosides, upregulate autophagy as a survival mechanism (Fujimoto et al., 2017; He et al., 2017), but then typically undergo apoptosis (Dinh et al., 2015; Pang et al., 2019). However, hair cells have also been found to die by necrosis (Owens et al., 2007; Dinh et al., 2015). Previous work in zebrafish V-ATPase mutant embryos found that cells in the retina aberrantly undergo apoptosis, which is readily detected by immunostaining for active Caspase 3 (Nuckels et al., 2009). To test whether *atp6v1f^−/−^* mutant hair cells also undergo apoptosis, we first used anti-cleaved Caspase 3 antibodies to detect active Caspase 3. At 2 dpf or 4 dpf, we observed little or no active Caspase 3 in wild-type or mutant neuromasts (Fig. 6A). However, retinas in the same 4 dpf mutant embryos contained Caspase 3-positive cells (Fig. 6A), as described (Nuckels et al., 2009). In additional positive-control experiments, Caspase 3-positive cells were detected in wild-type neuromasts treated with 400 µM neomycin, which activates caspase-dependent apoptosis of hair cells (Fig. 6A) (Harris et al., 2003; Uribe et al., 2015; Wiedenhoft et al., 2017). The absence of active Caspase 3 in *atp6v1f^−/−^* neuromasts suggested that the hair cell death is independent of the Caspase 3 pathway.

To further test the role of caspase activity in hair cell death in V-ATPase mutants, we used the pan-caspase inhibitor ZVAD-FMK that blocks apoptotic cell death. Previous work in zebrafish neuromasts has shown that ZVAD-FMK can prevent hair cell death induced by low doses of neomycin (Williams and Holder, 2000; Matsui et al., 2002). However, ZVAD-FMK treatments did not block hair cell death in *atp6v1f^−/−^* mutants (Fig. 6B,C), which provides additional evidence that V-ATPase mutant hair cells die independent of caspase activity. Next, live imaging of *atp6v1f^−/−^* mutants expressing the *Tg(myo6b:tdtomato)* transgene revealed that dying hair cells do not undergo the typical morphological changes associated with apoptosis, which include plasma membrane blebbing, cell shrinkage and formation of apoptotic bodies (Chen et al., 2018; Nirmala and Lopus, 2020). In contrast, dying mutant hair cells swell and rupture, which are changes associated with necrosis (Golstein and Kroemer, 2007; Nirmala and Lopus, 2020) (Fig. 6D; Movie 5). These morphological changes in mutant hair cells were similar to changes in wild-type hair cells treated with 10 µM CuSO_4_ (Movie 6), which is known to induce necrosis in neuromast hair cells (Olivari et al., 2008; Kasica-Jarosz et al., 2018). Together, these results indicate that hair cells undergo necrosis-like cell death that is independent of caspase activity.

### Loss of V-ATPase alters mitochondria in hair cells

We next wanted to begin to understand mechanistically how loss of V-ATPase activity leads to necrosis-like death of hair cells. V-ATPase is known to mediate several different cellular functions; therefore, we predicted that instead of one specific defect that triggers cell death, there may be several underlying problems that contribute to hair cells dying. Because loss of V-ATPase has previously been shown to increase reactive oxygen species (ROS) (Milgrom et al., 2007; Yokomakura et al., 2012), and elevated ROS levels can induce hair cell death (Esterberg et al., 2016), we first tested whether ROS levels are elevated in V-ATPase mutant hair cells. Surprisingly, using the fluorescent probe CellROX in live neuromasts as described (Esterberg et al., 2016; Razaghi et al., 2018), we did not detect an increase in ROS in *atp6v1f^−/−^* mutant hair cells, whereas quantification of ROS probe fluorescence indicated that hair cell ROS are decreased in mutants (Fig. S7). This suggests that loss of V-ATPase does not trigger an increase in ROS that contributes to hair cell death.

**Fig. 7.**
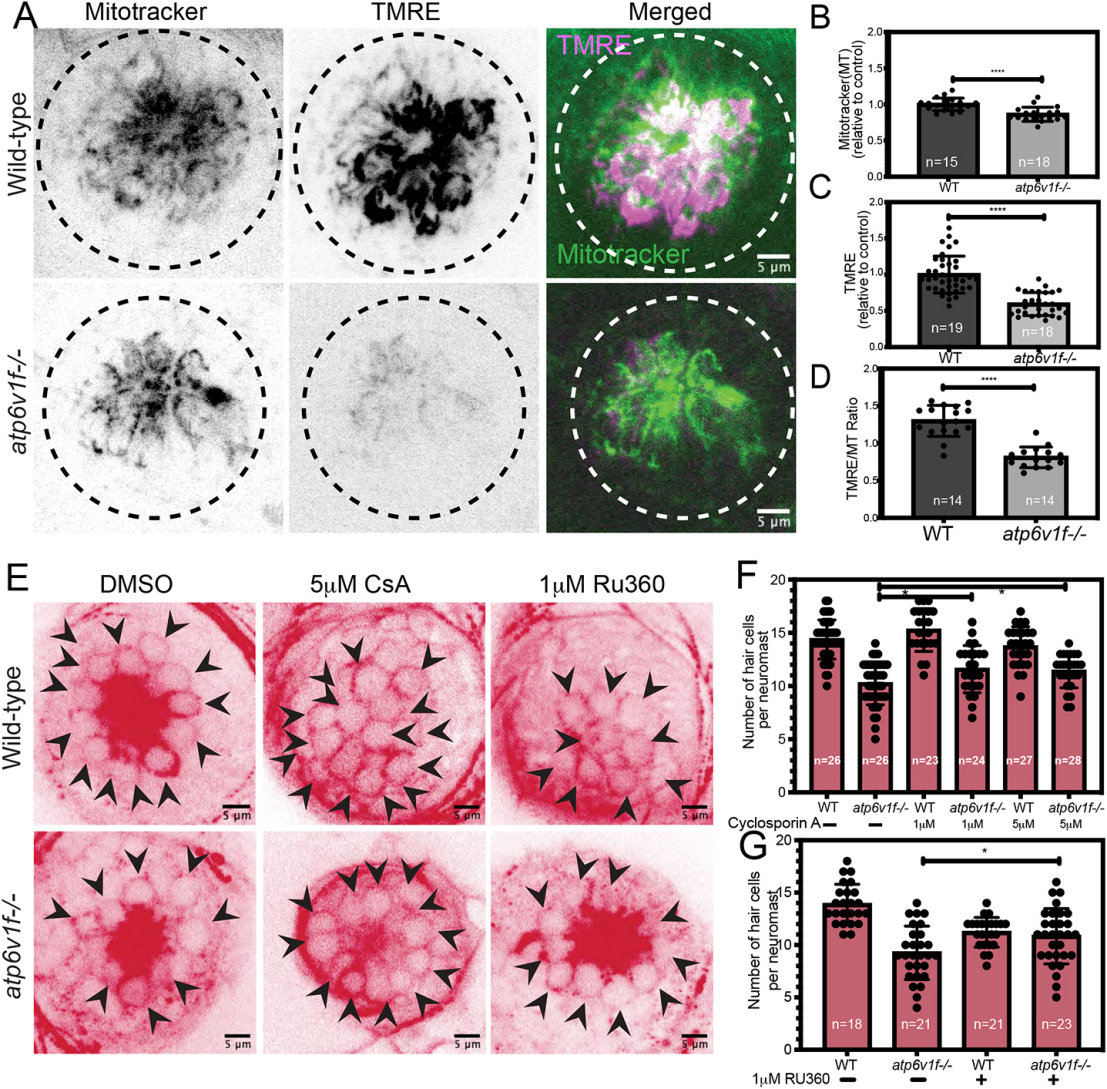
**Reduced mitochondrial membrane potential contributes to hair cell death in V-ATPase mutant neuromasts*.*** (A) The vital dyes MitoTracker and TMRE were used to assess mitochondrial mass and mitochondrial transmembrane potential, respectively, in live wild-type and *atp6v1f^−/−^* embryos at 4 dpf. Dashed line circles indicate the neuromast boundary. (B,C) Quantification of mean fluorescence intensity measurements per neuromast of MitoTracker (B) and TMRE (C) in wild-type and *atp6v1f^−/−^* embryos at 4 dpf. (D) Ratio of TMRE fluorescence intensity to MitoTracker. *n*=number of embryos. ****P<0.0001 by unpaired Student's *t*-test with Welch's correction. (E) Representative images of acetylated tubulin immunostaining of hair cells in wild-type and *atp6v1f^−/−^* embryos at 4 dpf after treatment with DMSO (vehicle control), CsA or RU360 from 2 dpf to 4 dpf. Arrowheads point to individual hair cells. (F,G) The number of hair cells per neuromast in control embryos and embryos treated with CsA (F) or RU360 (G). *n*=number of embryos. *P<0.04 by two-way ANOVA with Bonnferroni–Šidák post hoc test.

Because mitochondria are the primary source of cellular ROS (Kausar et al., 2018), the reduction in ROS in *atp6v1f^−/−^* mutant hair cells suggested a mitochondrial defect. Mitochondria are key players in cell death, and previous work has highlighted links between V-ATPase activity, lysosome function and mitochondrial health (Hughes and Gottschling, 2012; Bartel et al., 2019; Yambire et al., 2019). In mammalian cancer cell lines, pharmacological suppression of V-ATPase activity can result in depolarization of the mitochondrial membrane and apoptosis (Hong et al., 2006; De Milito et al., 2007; McHenry et al., 2010). In other contexts, loss of mitochondrial membrane potential (ΔΨm), which leads to mitochondrial dysfunction and loss of ATP production, is associated with necrosis (Navarro and Boveris, 2004; Karch and Molkentin, 2015; Jara et al., 2019). To analyze mitochondria in V-ATPase mutant hair cells, we co-stained living embryos with the vital dye MitoTracker to label the mitochondria and tetramethylrhodamine ester (TMRE) to visualize ΔΨm (Fig. 7A), as previously described in zebrafish neuromasts (Owens et al., 2007; Esterberg et al., 2014; Alassaf et al., 2019). At 4 dpf, we measured a decrease in overall MitoTracker fluorescence intensity between wild-type and *atp6v1f^−/−^* hair cell clusters (Fig. 7C), indicating reduced mitochondrial mass in mutants. Additionally, we found a more pronounced decrease in TMRE labeling in *atp6v1f^−/−^* hair cells compared to wild-type hair cells (Fig. 7B), which indicates lower ΔΨm. The ratio of TMRE/MitoTracker (Fig. 7D) indicates that reduced TMRE staining in the mutants is not only due to loss of mitochondria. Consistent with reduced ROS levels, these results indicate that V-ATPase loss leads to structural and functional defects in hair cell mitochondria.

### Modulating the mPTP improves survival of V-ATPase mutant hair cells

We next focused on whether mitochondrial membrane depolarization (reduced ΔΨm) contributes to V-ATPase mutant hair cell death. A decrease in ΔΨm can be due to prolonged opening of the mPTP. Inhibiting mPTP opening has been shown to prevent necrotic cell death of hepatocytes and cardiomyocytes (Kinnally et al., 2011; Kwong and Molkentin, 2015). To modulate mPTP, we treated embryos with the mPTP inhibitor cyclosporin A (CsA). CsA interacts with Cyclophilin D (CypD) protein, which regulates mPTP and has been successfully used in zebrafish hair cells to inhibit mPTP opening and increase ΔΨm (Esterberg et al., 2014). Immunostaining experiments revealed that CsA treatments increased the number of hair cells (Fig. 7E,F) and reduced the number of pyknotic nuclei (Fig. S8A) in *atp6v1f^−/−^* mutant embryos. This increase in hair cell survival was modest, but robust: a statistically significant increase in hair cell number was observed in mutant neuromasts in three independent experiments. These results suggest that inhibiting mPTP opening, and thereby modulating ΔΨm, can moderately improve hair cell survival in V-ATPase mutants.

As a second approach to modulate mPTP, we sought to test another regulator of mPTP opening. The primary stimulators that open the mPTP are thought to be ROS and calcium ions (Ca^2+^) (Bonora and Pinton, 2014). Because ROS levels are not elevated in *atp6v1f^−/−^* mutant hair cells (Fig. S7), we asked whether loss of V-ATPase activity alters Ca^2+^ handling in hair cells, which may impact mPTP function. Loss of V-ATPase has previously been associated with defects in Ca^2+^ homeostasis in diverse cell types (Förster and Kane, 2000; Christensen et al., 2002; López et al., 2005), including mammalian macrophages and platelets in which loss of V-ATPase increases cytoplasmic Ca^2+^ levels. We hypothesized that loss of V-ATPase may lead to a cytoplasmic Ca^2+^ overload that contributes to mPTP opening, mitochondrial defects and cell death. To functionally test this hypothesis, we inhibited the MCU with the Ruthenium Red derivative RU360 (Esterberg et al., 2014, 2016). The MCU is a Ca^2+^-activated Ca^2+^ channel that controls cytoplasmic Ca^2+^ entry into mitochondria. Prolonged elevation of Ca^2+^ levels in mitochondria results in mPTP opening and cell death (Bonora and Pinton, 2014). RU360 treatments from 2 dpf to 4 dpf were detrimental to wild-type neuromasts and led to reduced hair cell number (Fig. 7G). In contrast, RU360 increased hair cell number in *atp6v1f^−/−^* mutants (Fig. 7G). This suggests that Ca^2+^ handling is quite different between wild-type and mutant hair cells. The increase in mutant hair cell survival with RU360 treatments was comparable to that with CsA treatments that inhibit mPTP opening (Fig. 7F). The number of pyknotic nuclei was also reduced in *atp6v1f^−/−^* embryos treated with RU360, but this change did not reach statistical significance (Fig. S8B). Taken together, these results support a model in which loss of V-ATPase alters Ca^2+^ homeostasis and mPTP regulation, which leads to mitochondrial depolarization and dysfunction, which are contributing factors to the necrosis-like death of hair cells.

## DISCUSSION

In this study, we identify new cell-type-specific functions for V-ATPase *in vivo*. We show that V-ATPase activity is critical for the survival of mechanosensory hair cells, but not neighboring support cells, in zebrafish neuromasts. This is the first analysis of complete loss of V-ATPase activity in hair cells in any vertebrate. Multiple previous reports indicate that V-ATPase-associated cell death occurs via apoptosis (De Milito et al., 2007; Nuckels et al., 2009; You et al., 2009). However, loss of V-ATPase has also been found to cause caspase-independent cell death in cultured cell lines (Sasazawa et al., 2009; Graham et al., 2014; Yambire et al., 2019). Our work provides the first *in vivo* evidence that loss of V-ATPase activity leads to caspase-independent necrosis-like death of neuromast hair cells. In addition, our results provide a new *in vivo* case study that supports a growing body of evidence that links V-ATPase activity with mitochondrial function (De Milito et al., 2007; Graham et al., 2014). Our results suggest that loss of V-ATPase alters Ca^2+^ flow via the MCU, impacts regulation of the mPTP and disrupts ΔΨm in hair cells. Together, these findings suggest that mitochondrial dysfunction contributes to caspase-independent necrosis-like death of hair cells. We predict there is a vast scope of functions for V-ATPase activity in specific cell types that we are only beginning to uncover. Identifying these V-ATPase functions may help understand underlying causes of disease.

### Elucidating V-ATPase functions in specific cell types

Human gene mutations and studies using animal models have implicated V-ATPase activity in diverse functions in specific cells. Global knockout of the essential Atp6v0c subunit in mice results in severe developmental defects shortly after implantation (embryonic day 5.5-6.5) that lead to embryonic lethality (Inoue et al., 1999), but conditional knockouts have shed light on the functions of V-ATPase subunits in specific cell types. In addition, the zebrafish embryo has emerged as a useful model to understand V-ATPase functions. Zebrafish zygotic V-ATPase loss-of-function mutants, which complete embryogenesis and develop for several days due to maternal supply of subunit mRNA and/or protein, provide a platform for broad-based phenotyping and *in vivo* mechanistic studies. In addition to altered pigmentation, which likely reflects altered pH in melanosomes (Dooley et al., 2013), phenotypes in several other cell types have been analyzed in zebrafish V-ATPase mutants. Analysis of microphthalmia (small eyes) in mutants uncovered roles for V-ATPase activity regulating cell cycle exit of retinoblasts, proliferation of retinal stem cells and survival of developing neurons (Nuckels et al., 2009). In the developing zebrafish gastrointestinal tract, analysis of membrane protein trafficking in mutant intestinal epithelial cells revealed roles for V-ATPase-mediated trans-Golgi network luminal acidification in apical sorting and transport of membrane proteins (Levic et al., 2020). In a separate study, loss of V-ATPase was found to alter intrahepatic biliary duct formation, which was proposed to result from faulty protein sorting (EauClaire et al., 2012). Finally, CRISPR-mediated knockout of *atp6v1h* reduced bone formation, likely via upregulation of matrix metalloproteinases in osteoclast cells (Zhang et al., 2017). This study also identified a family of patients with decreased bone density that have a deleterious mutation in *ATP6V1H*, which highlights the use of zebrafish to model human disease. It becomes clear from these examples that V-ATPase has a broad spectrum of cell-type-specific functions.

Inhibiting V-ATPase activity during early zebrafish development altered positioning of the heart and gastrointestinal tract along the left-right body axis (Gokey et al., 2015). V-ATPase activity has been linked to left-right axis determination for many years (Adams et al., 2006), and, more recently, a copy number variants screen in patients with laterality defects (Cowan et al., 2016) identified a duplication of the *ATP6V1G1* suggesting a role for V-ATPase in human laterality. In zebrafish, V-ATPase gene knockdowns or small molecule inhibitors reduced proliferation – but not survival – of precursor cells that formed a left-right organizer that was reduced in size (Gokey et al., 2015). Similarly, neuromast size was reduced in the V-ATPase accessory gene *atp6ap1b* mutant embryos (Gokey et al., 2015). We predicted that the reduced size of neuromasts would be a consequence of reduced cell proliferation, as we observed in the left-right organizer. However, we found that loss of V-ATPase reduces the survival of mechanosensory hair cells in developing neuromasts. Moreover, we found that hair cells do not die via caspase-dependent apoptosis as described in the zebrafish retina (Nuckels et al., 2009), but rather undergo caspase-independent necrosis-like cell death. These novel findings highlight context-specific functions for V-ATPase activity during embryo development.

### V-ATPase and cell death

Functions for V-ATPase in cell survival and death are complex and context dependent. Because V-ATPase is involved in essential cellular processes, it makes sense that loss of V-ATPase would be lethal to cells. In many cell types, including the aforementioned zebrafish retinal neurons (Nuckels et al., 2009), murine osteoclasts (Okahashi et al., 1997), human cortical neurons (Hirose et al., 2019), and several human cancer cells such as leukemia (Zhang et al., 2015) and breast cancer (von Schwarzenberg et al., 2013) cells, absence or prolonged inhibition of V-ATPase activity leads to cell death via the apoptotic pathway. Indeed, inhibiting V-ATPase has been identified as a promising therapeutic target to kill cancer cells (Stransky et al., 2016). Several studies suggest that cancer cells are more susceptible to V-ATPase inhibition than non-cancer cells, which indicates that V-ATPase activity promotes cancer cell survival. On the other hand, V-ATPase activity is required for necrosis-like death of yeast cells and *Caenorhabditis elegans* neurons induced by stress (Syntichaki et al., 2005; Kim et al., 2012) and killing human cancer cell lines with CDK4/6 inhibitors (Hino et al., 2020), likely by creating an acidic environment that mediates specific cell death pathways. In these cases, V-ATPase loss or inhibition increases cell survival. These results reveal different functions for V-ATPase in different scenarios that promote cell survival or cell death.

Several studies indicate that loss of V-ATPase triggers apoptosis by increasing ROS. High levels of ROS result in mitochondrial membrane depolarization, release of cytochrome C and activation of apoptotic machinery that includes caspases (Lin and Beal, 2006; Du et al., 2015; Wang et al., 2017). However, we observed a decrease in CellROX signals in the *atp6v1f^−/−^* mutant hair cells, indicating that elevated levels of ROS is not a cause of hair cell death in these mutants. Work in hepatocellular carcinoma cell lines indicates that V-ATPase inhibition that causes mitochondrial impairment can hamper ROS generation (Bartel et al., 2019). Similarly, mitochondrial defects in V-ATPase mutant hair cells likely explain reduced ROS levels. In some *in vitro* studies, loss of V-ATPase has been associated with caspase-independent cell death. Treating cultured leukemia cell lines (Yuan et al., 2015) or hepatocellular carcinoma cell lines (Yan et al., 2016) with the V-ATPase inhibitor Bafilomycin A1 reduced proliferation and induced death in these cell types. In both studies, the mode of cell death was determined to be caspase independent because Caspase 3 activation was not observed and the pan-caspase inhibitor ZVAD-FMK had no effect. Additional experiments in leukemia cells uncovered mitochondrial membrane depolarization and release of apoptosis-inducing factor to the nucleus (Yuan et al., 2015). In contrast, death in hepatocellular carcinoma cell lines was proposed to involve autophagy and p38-MAPK pathways (Yan et al., 2016). It is important to note that these studies only tested the effect of Bafilomycin A1 on cancer cell death, so it is possible that cell death results from an off-target effect that is independent of V-ATPase. Using gene mutations, our results provide clear evidence that loss of V-ATPase activity can indeed lead to caspase-independent death of a specific cell type *in vivo*. However, the morphology of dying hair cells is consistent with necrotic-like cell death rather than apoptosis, and, in preliminary experiments, pharmacological inhibitors of autophagy or p38-MAPK did not change hair cell death in V-ATPase mutant zebrafish neuromasts. Instead, we found evidence that mitochondrial dysfunction contributes to hair cell death in V-ATPase mutants. Mitochondrial membrane depolarization is known to cause mitochondrial dysfunction, loss of ATP production and, ultimately, cell death. However, other consequences of mitochondrial depolarization, such as the potential for release of pro-death signals, may contribute to hair cell death. Because modulating ΔΨm only partially rescued hair cell survival in mutant neuromasts, we hypothesize that loss of V-ATPase induces additional defects that contribute to hair cell death. Future work is needed to test this hypothesis and potentially identify other mechanisms by which V-ATPase promotes hair cell survival.

### V-ATPase in hair cells

Hair cells are intriguing cell types that transduce mechanical stimuli into chemical signals; this includes detecting water flows by zebrafish neuromast and sounds in the mammalian inner ear. We report here that loss of V-ATPase leads to hair cell death in zebrafish neuromasts. Previous work has implicated V-ATPase in signaling pathways – including Notch and Wnt – that are known to be active in neuromasts. This made us wonder whether alterations in these pathways influenced the neuromast defects in V-ATPase mutants. First, loss of V-ATPase activity has been reported to reduce Notch signaling in diverse cell types, including *Drosophila* follicle cells and imaginal disc cells (Yan et al., 2009), rat retina cells (Valapala et al., 2013) and mouse neural precursors (Lange et al., 2011). V-ATPase-mediated pH regulation is thought to be critical for endocytosis, protease activation and protein degradation during Notch signal transduction (Sun-Wada and Wada, 2015). In mouse (Kiernan et al., 2005) and zebrafish (Itoh and Chitnis, 2001), blocking Notch signaling results in an increased number of hair cells at the expense of support cells. In contrast, we do not observe an increase in hair cells or a decrease in support cells in V-ATPase mutants. Second, blocking V-ATPase activity, in turn, blocks the endosomal processing and transmission of canonical Wnt signals (Buechling et al., 2010; Cruciat et al., 2010; Tuttle et al., 2014). Wnt signaling is known to promote cell proliferation in neuromasts, which is restricted by the Wnt antagonist Dickkopf (Dkk) proteins (Valdivia et al., 2011; Head et al., 2013; Wada et al., 2013). Inhibiting Wnt by over-expressing Dkk protein reduces neuromast size and hair cell number, which is similar to the phenotype seen in V-ATPase mutants. However, in contrast to V-ATPase mutants, blocking Wnt signaling was found to reduce cell proliferation in neuromasts, with no effect on cell survival (Wada et al., 2013). Although we do not rule out the possibility that subtle changes in Notch and/or Wnt signaling could contribute to V-ATPase mutant neuromast phenotypes, it is clear that the reduced survival of hair cells cannot be explained exclusively by alterations in one of these pathways.

In addition to modulating the Notch and Wnt pathways, V-ATPase regulates mTOR signaling. V-ATPase recruits mTOR complex 1 (mTORC1) to the lysosomal membrane, in response to amino acid stimulation or nutrient changes (Zoncu et al., 2011). Activation of mTORC1 at the lysosome promotes cellular growth. mTOR has not been implicated in hair cell development, but mTORC1 and mTORC2 are expressed in the mature mammalian cochlea. Reports of inhibiting mTOR with the small molecule rapamycin provide conflicting results on hair cell survival. *In vitro* treatments of cultured rat cochlear explants with rapamycin reduced hair cell number (Leitmeyer et al., 2015), whereas *in vivo* injections of rapamycin into rats protected hair cells from dying when exposed to the ototoxic drug cisplatin (Fang and Xiao, 2014). In our hands, inhibiting mTOR with rapamycin in zebrafish decreased hair cell number in both wild-type and *atp6v1f^−/−^* mutant neuromasts, but we did not observe an increase in the number of pyknotic nuclei in wild type or mutants. This suggests that mTOR signaling may promote hair cell formation (potentially via proliferation control) but does not impact hair cell survival during zebrafish neuromast development.

Our analysis of *atp6v1f^−/−^* mutants indicates that V-ATPase activity is required to maintain low pH in acidic cellular compartments in hair cells, which include endosomes, synaptic vesicles and lysosomes. The accumulation of autophagolysosomes in V-ATPase mutant hair cells indicates that lysosomal function is indeed impaired in these cells. Previous work has indicated that lysosomal function is linked to mitochondrial function. In yeast, genetic or pharmacological inhibition of V-ATPase reduced acidification of the vacuole (analogous to the lysosome) and led to an increase in cytosolic acidity, dissipation of ΔΨm and mitochondrial degradation (Hughes and Gottschling, 2012). More recently, inhibition of lysosomal acidification using V-ATPase inhibitors in mouse fibroblasts was shown to cause a reversible iron (Fe^2+^) deficiency that was linked to mitochondrial dysfunction and caspase-independent cell death (Yambire et al., 2019). However, in preliminary trials, iron supplementation did not prevent hair cell death in zebrafish V-ATPase mutants. In addition to Fe^2+^, intracellular Ca^2+^ levels, which are known to regulate mitochondria, are also modulated by lysosomes and V-ATPase activity (Lawrence and Zoncu, 2019). We found that using the drug RU360 to inhibit the MCU, which regulates Ca^2+^ flow into mitochondria, protects some hair cells in V-ATPase mutants. This is intriguing because RU360 treatments were harmful to wild-type neuromasts and reduced the number of hair cells. These results suggest a working model in which gradual loss of maternal V-ATPase activity in mutant neuromasts changes Ca^2+^ homeostasis, such that mitochondrial Ca^2+^ overload leads to mitochondrial depolarization and dysfunction, which contribute to caspase-independent necrosis-like death of hair cells.

Our findings identify zebrafish hair cells to be highly enriched for V-ATPase expression, and highly vulnerable to V-ATPase loss. This may be relevant on two biomedical fronts. First, although knockout mice can recapitulate sensorineural hearing loss found in patients with *ATP6V1B1* or *ATP6V0A4* mutations, exactly how V-ATPase functions in hearing is not fully understood. It is proposed that V-ATPase activity regulates the pH and ionic composition of the endolymphatic fluid in contact with hair cells in the inner ear, and when the homeostasis is disrupted this leads to loss of endocochlear potential, and an enlarged endolymph compartment and vestibular aqueduct (Lorente-Cánovas et al., 2013; Tian et al., 2017). Interestingly, hair cells appeared largely normal in deaf knockout mice at the stages analyzed (Dou et al., 2003; Hennings et al., 2012; Lorente-Cánovas et al., 2013). Our results indicate that hair cells depend on V-ATPase activity for survival, which raises the possibility that compromised V-ATPase activity in patients may sensitize hair cells to damage or stress, leading to an accumulation of hair cell death over time and, ultimately, resulting in hearing loss. A second consideration is the potential use of V-ATPase inhibitors as anti-cancer treatments. Some cancer cells are highly dependent on V-ATPase for survival and are more sensitive to V-ATPase inhibition than non-cancerous cells (Stransky et al., 2016), which makes V-ATPase inhibitors attractive candidates for chemotherapy. Similar to cancer cells, we found that neuromast hair cells are highly sensitive to loss of V-ATPase activity. This suggests that hair cell death may be a potential side effect of V-ATPase inhibitor treatments, similar to currently used platinum-based cancer drugs (cisplatin) and aminoglycoside antibiotics (Schacht et al., 2012). Future work is needed to test how V-ATPase inhibitor doses that kill cancer cells impact hair cells.

From the work presented here, we conclude that loss of V-ATPase activity induces caspase-independent necrosis-like death of hair cells in zebrafish neuromasts. Our work indicates that V-ATPase activity is necessary to maintain mitochondrial membrane polarization and mitochondrial health in hair cells, which are highly dependent on V-ATPase for survival relative to neighboring support cells in the neuromast. These results advance our understanding of cell-type-specific functions for V-ATPase activity, and provide insight into the underlying causes of sensorineural hearing loss and potentially other V-ATPase-associated diseases.

## MATERIALS AND METHODS

### Zebrafish strains

Zebrafish (*Danio rerio*) were maintained using standard protocols. Zebrafish embryos were collected from natural matings and staged according to Kimmel et al. (1995). Mutant strains used in this study include *atp6v1f^hi1988Tg^* and *atp6v1h^hi923Tg^*, which were obtained from the Zebrafish International Resource Center. Transgenic strains include *Tg(cldnb:lynGFP)* (Haas and Gilmour, 2006), *Tg(CMV:EGFP-map1lc3b)* (He et al., 2009), *Tg(myo6b:tdtomato)* and *Tg(scm1:GFP)* (Behra et al., 2009). All experiments were approved by State University of New York Upstate Medical University's Institutional Animal Care and Use Committee.

### Immunostaining

For fluorescent immunostaining experiments, embryos were fixed with 4% paraformaldehyde (Alfa Aesar) in phosphate-buffered saline+1% Tween 20 (PBST) overnight at 4°C. The next day, fix was removed, and the embryos were washed in PBST once for 15 min. This was followed by incubating the embryos in acetone for 8 min at −20°C and another wash with PBST for 15 min. The embryos were then blocked with PBS+10% bovine serum albumin (BSA) for 1 h at room temperature. Primary antibodies diluted in PBS+10% BSA were incubated with embryos overnight at 4°C. The embryos were then washed in PBST eight times for 15 min each. The embryos were again blocked and incubated with secondary antibodies in PBS+10% BSA overnight at 4°C, and washed eight times in PBST. Primary antibodies were as follows: mouse anti-acetylated tubulin, 1:200 (Sigma-Aldrich, T7451); mouse anti-Parvalbumin, 1:200 (Sigma-Aldrich, MAB1572); rabbit anti-Sox2, 1:200 (Abcam, ab97959); chicken anti-GFP, 1:200 (GeneTex, GTX13970); rabbit anti-Atp6v1a, 1:200 (Proteintech, 17115-1-AP); rabbit anti-Lamp1, 1:200 (Abcam, ab24170); mouse anti-BrdU, 1:200 (Santa Cruz Biotechnology, sc-32323); rabbit anti-pHH3, 1:200 (Cell Signaling Technology, 9701S); and rabbit anti-cleaved Caspase 3, 1:200 (Abcam, ab13847). Secondary antibodies were as follows: goat anti-mouse AlexaFluor 568, 1:200 (Thermo Fisher Scientific, A-11004); goat anti-rabbit AlexaFluor 488, 1:200 (Abcam, ab150077); and goat anti-chicken AlexaFluor 488, 1:200 (Invitrogen, A-11039). DAPI (1:500; Thermo Fisher Scientific, 62248) was used to stain nuclei.

### Spinning disc confocal microscopy

Immunostained or live zebrafish embryos were placed on their side on a 35 mm Petri dish with a cover glass bottom (MatTek) and immobilized in 2% low-melting-point agarose. For live imaging, embryos were anesthetized using 0.4% tricaine (Tokyo Chemical Industry, T0941). Images were captured using a Perkin-Elmer Ultra VIEW Vox spinning disc confocal microscope with a 40× objective lens and a Hamamatsu C9100-50 camera. Laser power and exposure times were kept the same between control and test groups for fluorescence intensity measurement experiments.

### Image processing and analysis

Confocal images were analyzed using Fiji (National Institutes of Health) software. Neuromast area was measured by drawing a circle around the neuromast using DAPI as a marker. Volumes of the aggregates of Lc3b-GFP were measured by using 3D Object Counter. Counting the number of hair cells and support cells in a neuromast was done by going through each *z*-slice of a stack of images through one neuromast. Representative images of neuromast hair cells and support cells were made by taking the maximum intensity projection of a few *z*-slices. Number of pyknotic nuclei was quantified using DAPI as a marker of nuclei using Cell Counter in Fiji. Both pHH3- and BrdU-positive cells were counted using these markers and DAPI used to identify nuclei using Cell Counter. TMRE and MitoTracker fluorescence mean intensities were measured by manually drawing a region of interest (ROI) around a cluster of hair cells of an individual neuromast in one channel and then copy-and-pasting the ROI in the other channel in Fiji.

### SEM

Embryos were fixed with 2.5% glutaraldehyde and 2 mM CaCl_2_ in 0.1 M cacodylate buffer for 1.5* *h. The embryos were then washed three times for 5 min each with cacodylate buffer. These embryos were then post-fixed with 1% osmium tetroxide and 4 mM CaCl_2_ in 80 mM cacodylate buffer for 10 min on ice, followed by three washes for 5 min each with MilliQ water. Embryos were then dehydrated using 50% to 100% graded ethanol (Kindt et al., 2012). Critical point drying was achieved using Tousimis Samdri-PVT-3b, sputter coated with palladium using Edwards sputter and coater. Images were collected using a JEOL JSM-IT100LA scanning electron microscope.

### RT-PCR

For qualitative analysis of V-ATPase subunit mRNA expression, total mRNA was isolated from *atp6v1f^−/−^*, *apt6v1h^−/−^* and wild-type sibling embryos using Trizol (Invitrogen, 15596018). cDNA was synthesized using the reverse transcriptase iScript kit (Bio-Rad), and PCR was used to amplify cDNAs of selected V-ATPase subunits and β-actin (primer sequences available upon request). PCR amplicons were scored as present or absent via agarose gel electrophoresis.

### Vital dyes

Live embryos were incubated in vital dyes diluted in embryo water at 28.5°C. After the incubation, live embryos were imaged using spinning disc confocal microscopy. Vital dyes used in this study were as follows: Lysotracker (Invitrogen, L7528), 100 nM for 20 min; CellROX (Invitrogen, C10444), 2 µM for 20 min; TMRE (Thermo Fisher Scientific, T669), 20 nM for 20 min; MitoTracker (Thermo Fisher Scientific, M7514), 100 nM for 10 min; EthD III (Biotium, 30065), diluted according to the manufacturer for 30 min; and Hoechst 33342 (NucBlue; Invitrogen, R37605), 8 µM for 30 min.

### BrdU assay

Embryos were incubated with 15% BrdU in embryo water for 1 h at 28.5°C. Embryos were then fixed with 4% paraformaldehyde overnight at 4°C, washed the next day with PBST (1% Tween 20), followed by incubation in 2N HCl for 30 min at 37°C (Cai et al., 2016). The immunostaining protocol was followed as mentioned above for anti-BrdU immunostaining and confocal imaging. The number of BrdU-positive cells per neuromast was determined by analyzing *z*-stacks.

### Pharmacological treatments

To induce hair cell death, embryos were incubated in 10, 200 or 400 µM neomycin sulfate (Sigma-Aldrich, 1458009) freshly prepared in embryo medium for 1* *h at 28.5°C as described (Ma et al., 2008; Cruz et al., 2015; Uribe et al., 2015), or 10 µM copper (ii) sulfate pentahydrate (Sigma-Aldrich, C8027) for 40 min at 28.5°C as described (Olivari et al., 2008). For all other drug treatments, embryos were incubated in embryo medium containing the drug or dimethyl sulfoxide (DMSO; vehicle control) from 2 dpf to 4 dpf at 28.5°C. The medium was refreshed daily. Drugs used included the following: 300 µM pan-caspase inhibitor ZVAD-FMK (Enzo Life Sciences, ALX-260-020-M001) as described (Williams and Holder, 2000; McNeill et al., 2007; Coffin et al., 2013), 1 µM or 5 µM CsA (Millipore Sigma, C3662) (Alassaf et al., 2019) and 1 µM Ru360 (Millipore Sigma, 557440).

### Statistical analysis

All statistical analyses were performed using GraphPad Prism 9. Graphs show ‘cleaned data’ wherever applicable and are devoid of outliers, as determined by GraphPad Prism following the ROUT method with Q=1%. *P*-values were calculated using unpaired Student's *t*-test with Welch's correction, when comparing means of two unpaired groups of data without assuming that they have identical standard deviation, or two-way ANOVA, when comparing means of multiple groups of data involving more than one variable, followed by post-hoc Bonnferroni–Šidák multiple comparisons because we compare selected sets of means, where the *P*-value needs to be less than alpha (0.05) to be considered statistically significant. All data are pooled from at least three independent experiments and represented as mean±s.d.

## Supplementary Material

Supplementary information

## References

[DMM048997C1] Abbas, Y. M., Wu, D., Bueler, S. A., Robinson, C. V. and Rubinstein, J. L. (2020). Structure of V-ATPase from the mammalian brain. *Science* 367, 1240-1246. 10.1126/science.aaz2924 32165585 PMC7324285

[DMM048997C2] Adams, D. S., Robinson, K. R., Fukumoto, T., Yuan, S., Albertson, R. C., Yelick, P., Kuo, L., McSweeney, M. and Levin, M. (2006). Early, H+-V-ATPase-dependent proton flux is necessary for consistent left-right patterning of non-mammalian vertebrates. *Development* 133, 1657-1671. 10.1242/dev.02341 16554361 PMC3136117

[DMM048997C3] Alassaf, M., Daykin, E. C., Mathiaparanam, J. and Wolman, M. A. (2019). Pregnancy-associated plasma protein-aa supports hair cell survival by regulating mitochondrial function. *eLife* 8, e47061. 10.7554/eLife.47061 31205004 PMC6594750

[DMM048997C4] Amsterdam, A., Nissen, R. M., Sun, Z., Swindell, E. C., Farrington, S. and Hopkins, N. (2004). Identification of 315 genes essential for early zebrafish development. *Proc. Natl. Acad. Sci. USA* 101, 12792-12797. 10.1073/pnas.0403929101 15256591 PMC516474

[DMM048997C5] Bartel, K., Pein, H., Popper, B., Schmitt, S., Janaki-Raman, S., Schulze, A., Lengauer, F., Koeberle, A., Werz, O., Zischka, H. et al. (2019). Connecting lysosomes and mitochondria - a novel role for lipid metabolism in cancer cell death. *Cell Commun. Signal.* 17, 87. 10.1186/s12964-019-0399-2 31358011 PMC6664539

[DMM048997C6] Behra, M., Bradsher, J., Sougrat, R., Gallardo, V., Allende, M. L. and Burgess, S. M. (2009). Phoenix is required for mechanosensory hair cell regeneration in the zebrafish lateral line. *PLoS Genet.* 5, e1000455. 10.1371/journal.pgen.1000455 19381250 PMC2662414

[DMM048997C7] Bonora, M. and Pinton, P. (2014). The mitochondrial permeability transition pore and cancer: molecular mechanisms involved in cell death. *Front. Oncol.* 4, 302. 10.3389/fonc.2014.00302 25478322 PMC4235083

[DMM048997C8] Buechling, T., Bartscherer, K., Ohkawara, B., Chaudhary, V., Spirohn, K., Niehrs, C. and Boutros, M. (2010). Wnt/Frizzled signaling requires dPRR, the Drosophila homolog of the prorenin receptor. *Curr. Biol.* 20, 1263-1268. 10.1016/j.cub.2010.05.028 20579883

[DMM048997C9] Cai, C., Lin, J., Sun, S. and He, Y. (2016). JNK inhibition inhibits lateral line neuromast hair cell development. *Front. Cell Neurosci.* 10, 19. 10.3389/fncel.2016.00019 26903805 PMC4742541

[DMM048997C10] Chen, Q., Kang, J. and Fu, C. (2018). The independence of and associations among apoptosis, autophagy, and necrosis. *Signal Transduct. Target. Ther.* 3, 18. 10.1038/s41392-018-0018-5 29967689 PMC6026494

[DMM048997C11] Christensen, K. A., Myers, J. T. and Swanson, J. A. (2002). pH-dependent regulation of lysosomal calcium in macrophages. *J. Cell Sci.* 115, 599-607. 10.1242/jcs.115.3.599 11861766

[DMM048997C12] Coffin, A. B., Williamson, K. L., Mamiya, A., Raible, D. W. and Rubel, E. W. (2013). Profiling drug-induced cell death pathways in the zebrafish lateral line. *Apoptosis* 18, 393-408. 10.1007/s10495-013-0816-8 23413197 PMC3627356

[DMM048997C13] Collins, M. P. and Forgac, M. (2018). Regulation of V-ATPase assembly in nutrient sensing and function of V-ATPases in breast cancer metastasis. *Front. Physiol.* 9, 902. 10.3389/fphys.2018.00902 30057555 PMC6053528

[DMM048997C14] Cotter, K., Stransky, L., McGuire, C. and Forgac, M. (2015). Recent insights into the structure, regulation, and function of the V-ATPases. *Trends Biochem. Sci.* 40, 611-622. 10.1016/j.tibs.2015.08.005 26410601 PMC4589219

[DMM048997C15] Cowan, J. R., Tariq, M., Shaw, C., Rao, M., Belmont, J. W., Lalani, S. R., Smolarek, T. A. and Ware, S. M. (2016). Copy number variation as a genetic basis for heterotaxy and heterotaxy-spectrum congenital heart defects. *Philos. Trans. R. Soc. B Biol. Sci.* 371, 20150406. 10.1098/rstb.2015.0406 PMC5104505 27821535

[DMM048997C16] Cruciat, C.-M., Ohkawara, B., Acebron, S. P., Karaulanov, E., Reinhard, C., Ingelfinger, D., Boutros, M. and Niehrs, C. (2010). Requirement of prorenin receptor and vacuolar H+-ATPase-mediated acidification for Wnt signaling. *Science* 327, 459-463. 10.1126/science.1179802 20093472

[DMM048997C17] Cruz, I. A., Kappedal, R., Mackenzie, S. M., Hailey, D. W., Hoffman, T. L., Schilling, T. F. and Raible, D. W. (2015). Robust regeneration of adult zebrafish lateral line hair cells reflects continued precursor pool maintenance. *Dev. Biol.* 402, 229-238. 10.1016/j.ydbio.2015.03.019 25869855 PMC4450121

[DMM048997C18] Davies, S. A., Goodwin, S. F., Kelly, D. C., Wang, Z., Sözen, M. A., Kaiser, K. and Dow, J. A. T. (1996). Analysis and inactivation of vha55, the gene encoding the vacuolar ATPase B-subunit in Drosophila melanogaster reveals a larval lethal phenotype. *J. Biol. Chem.* 271, 30677-30684. 10.1074/jbc.271.48.30677 8940044

[DMM048997C19] De Milito, A., Iessi, E., Logozzi, M., Lozupone, F., Spada, M., Marino, M. L., Federici, C., Perdicchio, M., Matarrese, P., Lugini, L. et al. (2007). Proton pump inhibitors induce apoptosis of human B-cell tumors through a caspase-independent mechanism involving reactive oxygen species. *Cancer Res.* 67, 5408-5417. 10.1158/0008-5472.CAN-06-4095 17545622

[DMM048997C20] Dinh, C. T., Goncalves, S., Bas, E., Van De Water, T. R. and Zine, A. (2015). Molecular regulation of auditory hair cell death and approaches to protect sensory receptor cells and/or stimulate repair following acoustic trauma. *Front. Cell Neurosci.* 9, 96. 10.3389/fncel.2015.00096 25873860 PMC4379916

[DMM048997C21] Dooley, C. M., Schwarz, H., Mueller, K. P., Mongera, A., Konantz, M., Neuhauss, S. C. F., Nüsslein-Volhard, C. and Geisler, R. (2013). Slc45a2 and V-ATPase are regulators of melanosomal pH homeostasis in zebrafish, providing a mechanism for human pigment evolution and disease. *Pigment Cell Melanoma Res.* 26, 205-217. 10.1111/pcmr.12053 23205854

[DMM048997C22] Dou, H., Finberg, K., Cardell, E. L., Lifton, R. and Choo, D. (2003). Mice lacking the B1 subunit of H^+^ -ATPase have normal hearing. *Hear. Res.* 180, 76-84. 10.1016/S0378-5955(03)00108-4 12782355

[DMM048997C23] Du, Z., Li, S., Liu, L., Yang, Q., Zhang, H. and Gao, C. (2015). NADPH oxidase 3associated oxidative stress and caspase 3dependent apoptosis in the cochleae of Dgalactoseinduced aged rats. *Mol. Med. Rep.* 12, 7883-7890. 10.3892/mmr.2015.4430 26498835 PMC4758280

[DMM048997C24] EauClaire, S. F., Cui, S., Ma, L., Matous, J., Marlow, F. L., Gupta, T., Burgess, H. A., Abrams, E. W., Kapp, L. D., Granato, M. et al. (2012). Mutations in vacuolar H^+^-ATPase subunits lead to biliary developmental defects in zebrafish. *Dev. Biol.* 365, 434-444. 10.1016/j.ydbio.2012.03.009 22465374 PMC3337356

[DMM048997C25] Einhorn, Z., Trapani, J. G., Liu, Q. and Nicolson, T. (2012). Rabconnectin3alpha promotes stable activity of the H^+^ pump on synaptic vesicles in hair cells. *J. Neurosci.* 32, 11144-11156. 10.1523/JNEUROSCI.1705-12.2012 22875945 PMC3428958

[DMM048997C26] Esterberg, R., Hailey, D. W., Rubel, E. W. and Raible, D. W. (2014). ER-mitochondrial calcium flow underlies vulnerability of mechanosensory hair cells to damage. *J. Neurosci.* 34, 9703-9719. 10.1523/JNEUROSCI.0281-14.2014 25031409 PMC4099547

[DMM048997C27] Esterberg, R., Linbo, T., Pickett, S. B., Wu, P., Ou, H. C., Rubel, E. W. and Raible, D. W. (2016). Mitochondrial calcium uptake underlies ROS generation during aminoglycoside-induced hair cell death. *J. Clin. Invest* 126, 3556-3566. 10.1172/JCI84939 27500493 PMC5004972

[DMM048997C28] Fang, B. and Xiao, H. (2014). Rapamycin alleviates cisplatin-induced ototoxicity in vivo. *Biochem. Biophys. Res. Commun.* 448, 443-447. 10.1016/j.bbrc.2014.04.123 24796670

[DMM048997C29] Förster, C. and Kane, P. M. (2000). Cytosolic Ca2+ homeostasis is a constitutive function of the V-ATPase in Saccharomyces cerevisiae. *J. Biol. Chem.* 275, 38245-38253. 10.1074/jbc.M006650200 10991947

[DMM048997C30] Froehlicher, M., Liedtke, A., Groh, K. J., Neuhauss, S. C. F., Segner, H. and Eggen, R. I. L. (2009). Zebrafish (Danio rerio) neuromast: promising biological endpoint linking developmental and toxicological studies. *Aquat. Toxicol.* 95, 307-319. 10.1016/j.aquatox.2009.04.007 19467721

[DMM048997C31] Fujimoto, C., Iwasaki, S., Urata, S., Morishita, H., Sakamaki, Y., Fujioka, M., Kondo, K., Mizushima, N. and Yamasoba, T. (2017). Autophagy is essential for hearing in mice. *Cell Death Dis.* 8, e2780. 10.1038/cddis.2017.194 28492547 PMC5520715

[DMM048997C32] Gillespie, P. G. and Müller, U. (2009). Mechanotransduction by hair cells: models, molecules, and mechanisms. *Cell* 139, 33-44. 10.1016/j.cell.2009.09.010 19804752 PMC2888516

[DMM048997C33] Gokey, J. J., Dasgupta, A. and Amack, J. D. (2015). The V-ATPase accessory protein Atp6ap1b mediates dorsal forerunner cell proliferation and left-right asymmetry in zebrafish. *Dev. Biol.* 407, 115-130. 10.1016/j.ydbio.2015.08.002 26254189 PMC4641761

[DMM048997C34] Golstein, P. and Kroemer, G. (2007). Cell death by necrosis: towards a molecular definition. *Trends Biochem. Sci.* 32, 37-43. 10.1016/j.tibs.2006.11.001 17141506

[DMM048997C35] Graham, R. M., Thompson, J. W. and Webster, K. A. (2014). Inhibition of the vacuolar ATPase induces Bnip3-dependent death of cancer cells and a reduction in tumor burden and metastasis. *Oncotarget* 5, 1162-1173. 10.18632/oncotarget.1699 24811485 PMC4012732

[DMM048997C36] Haas, P. and Gilmour, D. (2006). Chemokine signaling mediates self-organizing tissue migration in the zebrafish lateral line. *Dev. Cell* 10, 673-680. 10.1016/j.devcel.2006.02.019 16678780

[DMM048997C37] Harris, J. A., Cheng, A. G., Cunningham, L. L., MacDonald, G., Raible, D. W. and Rubel, E. W. (2003). Neomycin-induced hair cell death and rapid regeneration in the lateral line of zebrafish (*Danio rerio*). *J. Assoc. Res. Otolaryngol.* 4, 219-234. 10.1007/s10162-002-3022-x 12943374 PMC3202713

[DMM048997C38] He, C., Bartholomew, C. R., Zhou, W. and Klionsky, D. J. (2009). Assaying autophagic activity in transgenic GFP-Lc3 and GFP-Gabarap zebrafish embryos. *Autophagy* 5, 520-526. 10.4161/auto.5.4.7768 19221467 PMC2754832

[DMM048997C39] He, Z., Guo, L., Shu, Y., Fang, Q., Zhou, H., Liu, Y., Liu, D., Lu, L., Zhang, X., Ding, X. et al. (2017). Autophagy protects auditory hair cells against neomycin-induced damage. *Autophagy* 13, 1884-1904. 10.1080/15548627.2017.1359449 28968134 PMC5788479

[DMM048997C40] Head, J. R., Gacioch, L., Pennisi, M. and Meyers, J. R. (2013). Activation of canonical Wnt/β-catenin signaling stimulates proliferation in neuromasts in the zebrafish posterior lateral line. *Dev. Dyn.* 242, 832-846. 10.1002/dvdy.23973 23606225

[DMM048997C41] Hennings, J. C., Picard, N., Huebner, A. K., Stauber, T., Maier, H., Brown, D., Jentsch, T. J., Vargas-Poussou, R., Eladari, D. and Hübner, C. A. (2012). A mouse model for distal renal tubular acidosis reveals a previously unrecognized role of the V-ATPase a4 subunit in the proximal tubule. *EMBO Mol. Med.* 4, 1057-1071. 10.1002/emmm.201201527 22933323 PMC3491836

[DMM048997C42] Hernández, P. P., Olivari, F. A., Sarrazin, A. F., Sandoval, P. C. and Allende, M. L. (2007). Regeneration in zebrafish lateral line neuromasts: expression of the neural progenitor cell marker sox2 and proliferation-dependent and-independent mechanisms of hair cell renewal. *Dev. Neurobiol.* 67, 637-654. 10.1002/dneu.20386 17443814

[DMM048997C43] Hino, H., Iriyama, N., Kokuba, H., Kazama, H., Moriya, S., Takano, N., Hiramoto, M., Aizawa, S. and Miyazawa, K. (2020). Abemaciclib induces atypical cell death in cancer cells characterized by formation of cytoplasmic vacuoles derived from lysosomes. *Cancer Sci.* 111, 2132-2145. 10.1111/cas.14419 32304130 PMC7293084

[DMM048997C44] Hirose, T., Cabrera-Socorro, A., Chitayat, D., Lemonnier, T., Féraud, O., Cifuentes-Diaz, C., Gervasi, N., Mombereau, C., Ghosh, T., Stoica, L. et al. (2019). ATP6AP2 variant impairs CNS development and neuronal survival to cause fulminant neurodegeneration. *J. Clin. Invest* 129, 2145-2162. 10.1172/JCI79990 30985297 PMC6486358

[DMM048997C45] Ho, M. N., Hirata, R., Umemoto, N., Ohya, Y., Takatsuki, A., Stevens, T. H. and Anraku, Y. (1993). VMA13 encodes a 54-kDa vacuolar H(+)-ATPase subunit required for activity but not assembly of the enzyme complex in Saccharomyces cerevisiae. *J. Biol. Chem.* 268, 18286-18292. 10.1016/S0021-9258(17)46842-6 8349704

[DMM048997C46] Holliday, L. S. (2014). Vacuolar H^+^-ATPase: an essential multitasking enzyme in physiology and pathophysiology. *New J. Sci.* 2014, 675430. 10.1155/2014/675430

[DMM048997C47] Hong, J., Nakano, Y., Yokomakura, A., Ishihara, K., Kim, S., Kang, Y.-S. and Ohuchi, K. (2006). Nitric oxide production by the vacuolar-type (H^+^)-ATPase inhibitors bafilomycin A1 and concanamycin A and its possible role in apoptosis in RAW 264.7 cells. *J. Pharmacol. Exp. Ther.* 319, 672-681. 10.1124/jpet.106.109280 16895977

[DMM048997C48] Hou, L., Liu, K., Li, Y., Ma, S., Ji, X. and Liu, L. (2016). Necrotic pyknosis is a morphologically and biochemically distinct event from apoptotic pyknosis. *J. Cell Sci.* 129, 3084-3090. 10.1242/jcs.184374 27358477

[DMM048997C49] Hughes, A. L. and Gottschling, D. E. (2012). An early age increase in vacuolar pH limits mitochondrial function and lifespan in yeast. *Nature* 492, 261-265. 10.1038/nature11654 23172144 PMC3521838

[DMM048997C50] Inoue, H., Noumi, T., Nagata, M., Murakami, H. and Kanazawa, H. (1999). Targeted disruption of the gene encoding the proteolipid subunit of mouse vacuolar H^+^-ATPase leads to early embryonic lethality. *Biochim. Biophys. Acta (BBA) Bioenerg.* 1413, 130-138. 10.1016/S0005-2728(99)00096-1 10556625

[DMM048997C51] Itoh, M. and Chitnis, A. B. (2001). Expression of proneural and neurogenic genes in the zebrafish lateral line primordium correlates with selection of hair cell fate in neuromasts. *Mech. Dev.* 102, 263-266. 10.1016/S0925-4773(01)00308-2 11287207

[DMM048997C52] Jansen, E. J. R., Timal, S., Ryan, M., Ashikov, A., van Scherpenzeel, M., Graham, L. A., Mandel, H., Hoischen, A., Iancu, T. C., Raymond, K. et al. (2016). ATP6AP1 deficiency causes an immunodeficiency with hepatopathy, cognitive impairment and abnormal protein glycosylation. *Nat. Commun.* 7, 11600. 10.1038/ncomms11600 27231034 PMC4894975

[DMM048997C53] Jara, C., Torres, A. K., Olesen, M. A. and Tapia-Rojas, C. (2019). Mitochondrial dysfunction as a key event during aging: from synaptic failure to memory loss. In *Mitochondria* *and* *Brain Disord**ers*. IntechOpen. 10.5772/intechopen.88445

[DMM048997C54] Kane, P. M. (2007). The long physiological reach of the yeast vacuolar H+-ATPase. *J. Bioenerg. Biomembr.* 39, 415-421. 10.1007/s10863-007-9112-z 18000744 PMC2901503

[DMM048997C55] Karch, J. and Molkentin, J. D. (2015). Regulated necrotic cell death: the passive aggressive side of Bax and Bak. *Circ. Res.* 116, 1800-1809. 10.1161/CIRCRESAHA.116.305421 25999420 PMC4443748

[DMM048997C56] Karet, F. E., Finberg, K. E., Nelson, R. D., Nayir, A., Mocan, H., Sanjad, S. A., Rodriguez-Soriano, J., Santos, F., Cremers, C. W. R. J., Di Pietro, A. et al. (1999). Mutations in the gene encoding B1 subunit of H+-ATPase cause renal tubular acidosis with sensorineural deafness. *Nat. Genet.* 21, 84-90. 10.1038/5022 9916796

[DMM048997C57] Kasica-Jarosz, N., Podlasz, P. and Kaleczyc, J. (2018). Pituitary adenylate cyclase-activating polypeptide (PACAP-38) plays an inhibitory role against inflammation induced by chemical damage to zebrafish hair cells. *PLoS ONE* 13, e0198180. 10.1371/journal.pone.0198180 29856797 PMC5983416

[DMM048997C58] Kausar, S., Wang, F. and Cui, H. (2018). The role of mitochondria in reactive oxygen species generation and its implications for neurodegenerative diseases. *Cells* 7, 274. 10.3390/cells7120274 PMC6316843 30563029

[DMM048997C59] Kiernan, A. E., Cordes, R., Kopan, R., Gossler, A. and Gridley, T. (2005). The Notch ligands DLL1 and JAG2 act synergistically to regulate hair cell development in the mammalian inner ear. *Development* 132, 4353-4362. 10.1242/dev.02002 16141228

[DMM048997C60] Kim, H., Kim, A. and Cunningham, K. W. (2012). Vacuolar H+-ATPase (V-ATPase) promotes vacuolar membrane permeabilization and nonapoptotic death in stressed yeast. *J. Biol. Chem.* 287, 19029-19039. 10.1074/jbc.M112.363390 22511765 PMC3365936

[DMM048997C61] Kimmel, C. B., Ballard, W. W., Kimmel, S. R., Ullmann, B. and Schilling, T. F. (1995). Stages of embryonic development of the zebrafish. *Dev. Dyn.* 203, 253-310. 10.1002/aja.1002030302 8589427

[DMM048997C62] Kindt, K. S., Finch, G. and Nicolson, T. (2012). Kinocilia mediate mechanosensitivity in developing zebrafish hair cells. *Dev. Cell* 23, 329-341. 10.1016/j.devcel.2012.05.022 22898777 PMC3426295

[DMM048997C63] Kinnally, K. W., Peixoto, P. M., Ryu, S.-Y. and Dejean, L. M. (2011). Is mPTP the gatekeeper for necrosis, apoptosis, or both? *Biochim. Biophys. Acta (BBA) Mol. Cell Res.* 1813, 616-622. 10.1016/j.bbamcr.2010.09.013 PMC3050112 20888866

[DMM048997C64] Kornak, U., Schulz, A., Friedrich, W., Uhlhaas, S., Kremens, B., Voit, T., Hasan, C., Bode, U., Jentsch, T. J. and Kubisch, C. (2000). Mutations in the a3 subunit of the vacuolar H^+^-ATPase cause infantile malignant osteopetrosis. *Hum. Mol. Genet.* 9, 2059-2063. 10.1093/hmg/9.13.2059 10942435

[DMM048997C65] Kornak, U., Reynders, E., Dimopoulou, A., van Reeuwijk, J., Fischer, B., Rajab, A., Budde, B., Nürnberg, P., Foulquier, F., the ARCL Debré-type StudyGroup, Lefeber, D. et al. (2008). Impaired glycosylation and cutis laxa caused by mutations in the vesicular H+-ATPase subunit ATP6V0A2. *Nat. Genet.* 40, 32-34. 10.1038/ng.2007.45 18157129

[DMM048997C66] Kwong, J. Q. and Molkentin, J. D. (2015). Physiological and pathological roles of the mitochondrial permeability transition pore in the heart. *Cell Metab.* 21, 206-214. 10.1016/j.cmet.2014.12.001 25651175 PMC4616258

[DMM048997C67] Lange, C., Prenninger, S., Knuckles, P., Taylor, V., Levin, M. and Calegari, F. (2011). The H^+^ vacuolar ATPase maintains neural stem cells in the developing mouse cortex. *Stem Cells Dev.* 20, 843-850. 10.1089/scd.2010.0484 21126173 PMC3128780

[DMM048997C68] Lawrence, R. E. and Zoncu, R. (2019). The lysosome as a cellular centre for signalling, metabolism and quality control. *Nat. Cell Biol.* 21, 133-142. 10.1038/s41556-018-0244-7 30602725

[DMM048997C69] Leitmeyer, K., Glutz, A., Radojevic, V., Setz, C., Huerzeler, N., Bumann, H., Bodmer, D. and Brand, Y. (2015). Inhibition of mTOR by rapamycin results in auditory hair cell damage and decreased spiral ganglion neuron outgrowth and neurite formation in vitro. *Biomed. Res. Int.* 2015, 925890. 10.1155/2015/925890 25918725 PMC4395993

[DMM048997C70] Levic, D. S., Ryan, S., Marjoram, L., Honeycutt, J., Bagwell, J. and Bagnat, M. (2020). Distinct roles for luminal acidification in apical protein sorting and trafficking in zebrafish. *J. Cell Biol.* 219, e201908225. 10.1083/jcb.201908225 32328632 PMC7147097

[DMM048997C71] Lin, M. T. and Beal, M. F. (2006). Mitochondrial dysfunction and oxidative stress in neurodegenerative diseases. *Nature* 443, 787-795. 10.1038/nature05292 17051205

[DMM048997C72] López, J. J., Camello-Almaraz, C., Pariente, J. A., Salido, G. M. and Rosado, J. A. (2005). Ca2+ accumulation into acidic organelles mediated by Ca2+- and vacuolar H+-ATPases in human platelets. *Biochem. J.* 390, 243-252. 10.1042/BJ20050168 15847604 PMC1188269

[DMM048997C73] Lopez-Schier, H. and Hudspeth, A. J. (2005). Supernumerary neuromasts in the posterior lateral line of zebrafish lacking peripheral glia. *Proc. Natl. Acad. Sci. USA* 102, 1496-1501. 10.1073/pnas.0409361102 15677337 PMC547829

[DMM048997C74] Lorente-Cánovas, B., Ingham, N., Norgett, E. E., Golder, Z. J., Karet Frankl, F. E. and Steel, K. P. (2013). Mice deficient in H+-ATPase a4 subunit have severe hearing impairment associated with enlarged endolymphatic compartments within the inner ear. *Dis. Model Mech.* 6, 434-442. 10.1242/dmm.010645 23065636 PMC3597025

[DMM048997C75] Lush, M. E. and Piotrowski, T. (2014). Sensory hair cell regeneration in the zebrafish lateral line. *Dev. Dyn.* 243, 1187-1202. 10.1002/dvdy.24167 25045019 PMC4177345

[DMM048997C76] Ma, E. Y., Rubel, E. W. and Raible, D. W. (2008). Notch signaling regulates the extent of hair cell regeneration in the zebrafish lateral line. *J. Neurosci.* 28, 2261-2273. 10.1523/JNEUROSCI.4372-07.2008 18305259 PMC6671837

[DMM048997C77] Mangieri, L. R., Mader, B. J., Thomas, C. E., Taylor, C. A., Luker, A. M., Tse, T. E., Huisingh, C. and Shacka, J. J. (2014). ATP6V0C knockdown in neuroblastoma cells alters autophagy-lysosome pathway function and metabolism of proteins that accumulate in neurodegenerative disease. *PLoS ONE* 9, e93257. 10.1371/journal.pone.0093257 24695574 PMC3973706

[DMM048997C78] Marshansky, V. and Futai, M. (2008). The V-type H+-ATPase in vesicular trafficking: targeting, regulation and function. *Curr. Opin. Cell Biol.* 20, 415-426. 10.1016/j.ceb.2008.03.015 18511251 PMC7111286

[DMM048997C79] Marshansky, V., Rubinstein, J. L. and Grüber, G. (2014). Eukaryotic V-ATPase: novel structural findings and functional insights. *Biochim. Biophys. Acta (BBA) Bioener.* 1837, 857-879. 10.1016/j.bbabio.2014.01.018 24508215

[DMM048997C80] Matsui, J. I., Ogilvie, J. M. and Warchol, M. E. (2002). Inhibition of caspases prevents ototoxic and ongoing hair cell death. *J. Neurosci.* 22, 1218-1227. 10.1523/JNEUROSCI.22-04-01218.2002 11850449 PMC6757575

[DMM048997C81] Mauvezin, C., Nagy, P., Juhász, G. and Neufeld, T. P. (2015). Autophagosome-lysosome fusion is independent of V-ATPase-mediated acidification. *Nat. Commun.* 6, 7007. 10.1038/ncomms8007 25959678 PMC4428688

[DMM048997C82] McHenry, P., Wang, W.-L., Devitt, E., Kluesner, N., Davisson, V. J., McKee, E., Schweitzer, D., Helquist, P. and Tenniswood, M. (2010). Iejimalides A and B inhibit lysosomal vacuolar H+-ATPase (V-ATPase) activity and induce S-phase arrest and apoptosis in MCF-7 cells. *J. Cell Biochem.* 109, 634-642. 10.1002/jcb.22438 20039309

[DMM048997C83] McNeill, M. S., Paulsen, J., Bonde, G., Burnight, E., Hsu, M.-Y. and Cornell, R. A. (2007). Cell death of melanophores in zebrafish trpm7 mutant embryos depends on melanin synthesis. *J. Invest Dermatol.* 127, 2020-2030. 10.1038/sj.jid.5700710 17290233

[DMM048997C84] Milgrom, E., Diab, H., Middleton, F. and Kane, P. M. (2007). Loss of vacuolar proton-translocating ATPase activity in yeast results in chronic oxidative stress. *J. Biol. Chem.* 282, 7125-7136. 10.1074/jbc.M608293200 17215245

[DMM048997C85] Montalbano, G., Capillo, G., Laurà, R., Abbate, F., Levanti, M., Guerrera, M. C., Ciriaco, E. and Germanà, A. (2018). Neuromast hair cells retain the capacity of regeneration during heavy metal exposure. *Ann. Anat. Anatomischer Anzeiger* 218, 183-189. 10.1016/j.aanat.2018.03.007 29719206

[DMM048997C86] Nakamura, N., Matsuura, A., Wada, Y. and Ohsumi, Y. (1997). Acidification of vacuoles is required for autophagic degradation in the yeast, Saccharomyces cerevisiae. *J. Biochem.* 121, 338-344. 10.1093/oxfordjournals.jbchem.a021592 9089409

[DMM048997C87] Navarro, A. and Boveris, A. (2004). Rat brain and liver mitochondria develop oxidative stress and lose enzymatic activities on aging. *Am. J. Physiol. Regul. Integr. Comp. Physiol.* 287, R1244-R1249. 10.1152/ajpregu.00226.2004 15271654

[DMM048997C88] Nelson, H., Mandiyan, S. and Nelson, N. (1994). The Saccharomyces cerevisiae VMA7 gene encodes a 14-kDa subunit of the vacuolar H(+)-ATPase catalytic sector. *J. Biol. Chem.* 269, 24150-24155. 10.1016/S0021-9258(19)51061-4 7929071

[DMM048997C89] Nicolson, T. (2005). The genetics of hearing and balance in zebrafish. *Annu. Rev. Genet.* 39, 9-22. 10.1146/annurev.genet.39.073003.105049 16285850

[DMM048997C90] Nirmala, J. G. and Lopus, M. (2020). Cell death mechanisms in eukaryotes. *Cell Biol. Toxicol.* 36, 145-164. 10.1007/s10565-019-09496-2 31820165

[DMM048997C91] Nishi, T. and Forgac, M. (2002). The vacuolar (H+)-ATPases — nature's most versatile proton pumps. *Nat. Rev. Mol. Cell Biol.* 3, 94-103. 10.1038/nrm729 11836511

[DMM048997C92] Norgett, E. E., Golder, Z. J., Lorente-Canovas, B., Ingham, N., Steel, K. P. and Karet Frankl, F. E. (2012). Atp6v0a4 knockout mouse is a model of distal renal tubular acidosis with hearing loss, with additional extrarenal phenotype. *Proc. Natl. Acad. Sci. USA* 109, 13775-13780. 10.1073/pnas.1204257109 22872862 PMC3427075

[DMM048997C93] Nuckels, R. J., Ng, A., Darland, T. and Gross, J. M. (2009). The vacuolar-ATPase complex regulates retinoblast proliferation and survival, photoreceptor morphogenesis, and pigmentation in the zebrafish eye. *Invest. Ophthalmol. Vis. Sci.* 50, 893-905. 10.1167/iovs.08-2743 18836173

[DMM048997C94] Okahashi, N., Nakamura, I., Jimi, E., Koide, M., Suda, T. and Nishihara, T. (1997). Specific inhibitors of vacuolar H^+^-ATPase trigger apoptotic cell death of osteoclasts. *J. Bone Miner. Res.* 12, 1116-1123. 10.1359/jbmr.1997.12.7.1116 9200012

[DMM048997C95] Olivari, F. A., Hernández, P. P. and Allende, M. L. (2008). Acute copper exposure induces oxidative stress and cell death in lateral line hair cells of zebrafish larvae. *Brain Res.* 1244, 1-12. 10.1016/j.brainres.2008.09.050 18848822

[DMM048997C96] Owens, K. N., Cunningham, D. E., MacDonald, G., Rubel, E. W., Raible, D. W. and Pujol, R. (2007). Ultrastructural analysis of aminoglycoside-induced hair cell death in the zebrafish lateral line reveals an early mitochondrial response. *J. Comp. Neurol.* 502, 522-543. 10.1002/cne.21345 17394157

[DMM048997C97] Pang, J., Xiong, H., Zhan, T., Cheng, G., Jia, H., Ye, Y., Su, Z., Chen, H., Lin, H., Lai, L. et al. (2019). Sirtuin 1 and autophagy attenuate cisplatin-induced hair cell death in the mouse cochlea and zebrafish lateral line. *Front. Cell Neurosci.* 12, 515. 10.3389/fncel.2018.00515 30692914 PMC6339946

[DMM048997C98] Parzych, K. R. and Klionsky, D. J. (2014). An overview of autophagy: morphology, mechanism, and regulation. *Antioxid. Redox Signal.* 20, 460-473. 10.1089/ars.2013.5371 23725295 PMC3894687

[DMM048997C99] Pickett, S. B. and Raible, D. W. (2019). Water waves to sound waves: using Zebrafish to explore hair cell biology. *J. Assoc. Res. Otolaryngol.* 20, 1-19. 10.1007/s10162-018-00711-1 30635804 PMC6364261

[DMM048997C100] Razaghi, B., Steele, S. L., Prykhozhij, S. V., Stoyek, M. R., Hill, J. A., Cooper, M. D., McDonald, L., Lin, W., Daugaard, M., Crapoulet, N. et al. (2018). hace1 Influences zebrafish cardiac development via ROS-dependent mechanisms. *Dev. Dyn.* 247, 289-303. 10.1002/dvdy.24600 29024245

[DMM048997C101] Saha, S., Panigrahi, D. P., Patil, S. and Bhutia, S. K. (2018). Autophagy in health and disease: a comprehensive review. *Biomed. Pharmacother.* 104, 485-495. 10.1016/j.biopha.2018.05.007 29800913

[DMM048997C102] Sarrazin, A. F., Villablanca, E. J., Nuñez, V. A., Sandoval, P. C., Ghysen, A. and Allende, M. L. (2006). Proneural gene requirement for hair cell differentiation in the zebrafish lateral line. *Dev. Biol.* 295, 534-545. 10.1016/j.ydbio.2006.03.037 16678150

[DMM048997C103] Sasazawa, Y., Futamura, Y., Tashiro, E. and Imoto, M. (2009). Vacuolar H^+^-ATPase inhibitors overcome Bcl-xL-mediated chemoresistance through restoration of a caspase-independent apoptotic pathway. *Cancer Sci.* 100, 1460-1467. 10.1111/j.1349-7006.2009.01194.x 19459857 PMC11159986

[DMM048997C104] Schacht, J., Talaska, A. E. and Rybak, L. P. (2012). Cisplatin and aminoglycoside antibiotics: hearing loss and its prevention. *Anat. Rec. Adv. Integr. Anat. Evol. Biol.* 295, 1837-1850. 10.1002/ar.22578 PMC3596108 23045231

[DMM048997C105] Stover, E. H., Borthwick, K. J., Bavalia, C., Eady, N., Fritz, D. M., Rungroj, N., Giersch, A. B. S., Morton, C. C., Axon, P. R., Akil, I. et al. (2002). Novel ATP6V1B1 and ATP6V0A4 mutations in autosomal recessive distal renal tubular acidosis with new evidence for hearing loss. *J. Med. Genet.* 39, 796-803. 10.1136/jmg.39.11.796 12414817 PMC1735017

[DMM048997C106] Stransky, L., Cotter, K. and Forgac, M. (2016). The function of V-ATPases in cancer. *Physiol. Rev.* 96, 1071-1091. 10.1152/physrev.00035.2015 27335445 PMC4982037

[DMM048997C107] Subasioglu Uzak, A., Cakar, N., Comak, E., Yalcinkaya, F. and Tekin, M. (2013). ATP6V1B1 mutations in distal renal tubular acidosis and sensorineural hearing loss: clinical and genetic spectrum of five families. *Ren. Fail.* 35, 1281-1284. 10.3109/0886022X.2013.824362 23923981 PMC5483946

[DMM048997C108] Sun-Wada, G.-H. and Wada, Y. (2015). Role of vacuolar-type proton ATPase in signal transduction. *Biochim. Biophys. Acta (BBA) Bioener.* 1847, 1166-1172. 10.1016/j.bbabio.2015.06.010 26072192

[DMM048997C109] Sun-Wada, G.-H., Murata, Y., Yamamoto, A., Kanazawa, H., Wada, Y. and Futai, M. (2000). Acidic endomembrane organelles are required for mouse postimplantation development. *Dev. Biol.* 228, 315-325. 10.1006/dbio.2000.9963 11112332

[DMM048997C110] Syntichaki, P., Samara, C. and Tavernarakis, N. (2005). The vacuolar H^+^ -ATPase mediates intracellular acidification required for neurodegeneration in C. elegans. *Curr. Biol.* 15, 1249-1254. 10.1016/j.cub.2005.05.057 16005300

[DMM048997C111] Tian, C., Gagnon, L. H., Longo-Guess, C., Korstanje, R., Sheehan, S. M., Ohlemiller, K. K., Schrader, A. D., Lett, J. M. and Johnson, K. R. (2017). Hearing loss without overt metabolic acidosis in ATP6V1B1 deficient MRL mice, a new genetic model for non-syndromic deafness with enlarged vestibular aqueducts. *Hum. Mol. Genet.* 26, 3722-3735. 10.1093/hmg/ddx257 28934385 PMC5886195

[DMM048997C112] Toro, C., Trapani, J. G., Pacentine, I., Maeda, R., Sheets, L., Mo, W. and Nicolson, T. (2015). Dopamine modulates the activity of sensory hair cells. *J. Neurosci.* 35, 16494-16503. 10.1523/JNEUROSCI.1691-15.2015 26674873 PMC4679827

[DMM048997C113] Tuttle, A. M., Hoffman, T. L. and Schilling, T. F. (2014). Rabconnectin-3a regulates vesicle endocytosis and canonical Wnt signaling in zebrafish neural crest migration. *PLoS Biol.* 12, e1001852. 10.1371/journal.pbio.1001852 24802872 PMC4011682

[DMM048997C114] Uribe, P. M., Kawas, L. H., Harding, J. W. and Coffin, A. B. (2015). Hepatocyte growth factor mimetic protects lateral line hair cells from aminoglycoside exposure. *Front. Cell Neurosci.* 9, 3. 10.3389/fncel.2015.00003 25674052 PMC4309183

[DMM048997C115] Valapala, M., Hose, S., Gongora, C., Dong, L., Wawrousek, E. F., Samuel Zigler, J., Jr. and Sinha, D. (2013). Impaired endolysosomal function disrupts Notch signalling in optic nerve astrocytes. *Nat. Commun.* 4, 1629. 10.1038/ncomms2624 23535650 PMC3718029

[DMM048997C116] Valdivia, L. E., Young, R. M., Hawkins, T. A., Stickney, H. L., Cavodeassi, F., Schwarz, Q., Pullin, L. M., Villegas, R., Moro, E., Argenton, F. et al. (2011). Lef1-dependent Wnt/β-catenin signalling drives the proliferative engine that maintains tissue homeostasis during lateral line development. *Development* 138, 3931-3941. 10.1242/dev.062695 21862557 PMC3160090

[DMM048997C117] Van Trump, W. J. and McHenry, M. J. (2008). The morphology and mechanical sensitivity of lateral line receptors in zebrafish larvae (Danio rerio). *J. Exp. Biol.* 211, 2105-2115. 10.1242/jeb.016204 18552300

[DMM048997C118] Vargas-Poussou, R., Houillier, P., Le Pottier, N., Strompf, L., Loirat, C., Baudouin, V., Macher, M.-A., Déchaux, M., Ulinski, T., Nobili, F. et al. (2006). Genetic investigation of autosomal recessive distal renal tubular acidosis: evidence for early sensorineural hearing loss associated with mutations in the ATP6V0A4 gene. *J. Am. Soc. Nephrol.* 17, 1437-1443. 10.1681/ASN.2005121305 16611712

[DMM048997C119] Vasanthakumar, T. and Rubinstein, J. L. (2020). Structure and roles of V-type ATPases. *Trends Biochem. Sci.* 45, 295-307. 10.1016/j.tibs.2019.12.007 32001091

[DMM048997C120] von Schwarzenberg, K., Wiedmann, R. M., Oak, P., Schulz, S., Zischka, H., Wanner, G., Efferth, T., Trauner, D. and Vollmar, A. M. (2013). Mode of cell death induction by pharmacological vacuolar H+-ATPase (V-ATPase) inhibition. *J. Biol. Chem.* 288, 1385-1396. 10.1074/jbc.M112.412007 23168408 PMC3543021

[DMM048997C121] Wada, H., Ghysen, A., Asakawa, K., Abe, G., Ishitani, T. and Kawakami, K. (2013). Wnt/Dkk negative feedback regulates sensory organ size in zebrafish. *Curr. Biol.* 23, 1559-1565. 10.1016/j.cub.2013.06.035 23891113

[DMM048997C122] Wang, S., Wang, J., Zhao, A. and Li, J. (2017). SIRT1 activation inhibits hyperglycemia-induced apoptosis by reducing oxidative stress and mitochondrial dysfunction in human endothelial cells. *Mol. Med. Rep.* 16, 3331-3338. 10.3892/mmr.2017.7027 28765962

[DMM048997C123] Wang, L., Wu, D., Robinson, C. V., Wu, H. and Fu, T.-M. (2020). Structures of a complete human V-ATPase reveal mechanisms of its assembly. *Mol. Cell* 80, 501-511.e3. 10.1016/j.molcel.2020.09.029 33065002 PMC7655608

[DMM048997C124] Whitfield, T. T. (2002). Zebrafish as a model for hearing and deafness. *J. Neurobiol.* 53, 157-171. 10.1002/neu.10123 12382273

[DMM048997C125] Wiedenhoft, H., Hayashi, L. and Coffin, A. B. (2017). PI3K and inhibitor of apoptosis proteins modulate gentamicin- induced hair cell death in the zebrafish lateral line. *Front. Cell Neurosci.* 11, 326. 10.3389/fncel.2017.00326 29093665 PMC5651234

[DMM048997C126] Williams, J. A. and Holder, N. (2000). Cell turnover in neuromasts of zebrafish larvae. *Hear. Res.* 143, 171-181. 10.1016/S0378-5955(00)00039-3 10771194

[DMM048997C127] Xia, Y., Liu, N., Xie, X., Bi, G., Ba, H., Li, L., Zhang, J., Deng, X., Yao, Y., Tang, Z. et al. (2019). The macrophage-specific V-ATPase subunit ATP6V0D2 restricts inflammasome activation and bacterial infection by facilitating autophagosome-lysosome fusion. *Autophagy* 15, 960-975. 10.1080/15548627.2019.1569916 30681394 PMC6526827

[DMM048997C128] Yambire, K. F., Rostosky, C., Watanabe, T., Pacheu-Grau, D., Torres-Odio, S., Sanchez-Guerrero, A., Senderovich, O., Meyron-Holtz, E. G., Milosevic, I., Frahm, J. et al. (2019). Impaired lysosomal acidification triggers iron deficiency and inflammation in vivo. *eLife* 8, e51031. 10.7554/eLife.51031 31793879 PMC6917501

[DMM048997C129] Yan, Y., Denef, N. and Schüpbach, T. (2009). The vacuolar proton pump, V-ATPase, is required for notch signaling and endosomal trafficking in *Drosophila*. *Dev. Cell* 17, 387-402. 10.1016/j.devcel.2009.07.001 19758563 PMC2758249

[DMM048997C130] Yan, Y., Jiang, K., Liu, P., Zhang, X., Dong, X., Gao, J., Liu, Q., Barr, M. P., Zhang, Q., Hou, X. et al. (2016). Bafilomycin A1 induces caspase-independent cell death in hepatocellular carcinoma cells via targeting of autophagy and MAPK pathways. *Sci. Rep.* 6, 37052. 10.1038/srep37052 27845389 PMC5109251

[DMM048997C131] Yokomakura, A., Hong, J., Ohuchi, K., Oh, S.-E., Lee, J.-Y., Mano, N., Takahashi, T., Hwang, G.-W. and Naganuma, A. (2012). Increased production of reactive oxygen species by the vacuolar-type (H^+^)-ATPase inhibitors bafilomycin A1 and concanamycin A in RAW 264 cells. *J. Toxicol. Sci.* 37, 1045-1048. 10.2131/jts.37.1045 23038011

[DMM048997C132] You, H., Jin, J., Shu, H., Yu, B., De Milito, A., Lozupone, F., Deng, Y., Tang, N., Yao, G., Fais, S. et al. (2009). Small interfering RNA targeting the subunit ATP6L of proton pump V-ATPase overcomes chemoresistance of breast cancer cells. *Cancer Lett.* 280, 110-119. 10.1016/j.canlet.2009.02.023 19299075

[DMM048997C133] Yuan, N., Song, L., Zhang, S., Lin, W., Cao, Y., Xu, F., Fang, Y., Wang, Z., Zhang, H., Li, X. et al. (2015). Bafilomycin A1 targets both autophagy and apoptosis pathways in pediatric B-cell acute lymphoblastic leukemia. *Haematologica* 100, 345-356. 10.3324/haematol.2014.113324 25512644 PMC4349273

[DMM048997C134] Zhang, S., Schneider, L. S., Vick, B., Grunert, M., Jeremias, I., Menche, D., Müller, R., Vollmar, A. M. and Liebl, J. (2015). Anti-leukemic effects of the V-ATPase inhibitor Archazolid A. *Oncotarget* 6, 43508-43528. 10.18632/oncotarget.6180 26496038 PMC4791247

[DMM048997C135] Zhang, Y., Huang, H., Zhao, G., Yokoyama, T., Vega, H., Huang, Y., Sood, R., Bishop, K., Maduro, V., Accardi, J. et al. (2017). ATP6V1H deficiency impairs bone development through activation of MMP9 and MMP13. *PLoS Genet.* 13, e1006481. 10.1371/journal.pgen.1006481 28158191 PMC5291374

[DMM048997C136] Zoncu, R., Bar-Peled, L., Efeyan, A., Wang, S., Sancak, Y. and Sabatini, D. M. (2011). mTORC1 senses lysosomal amino acids through an inside-out mechanism that requires the vacuolar H^+^-ATPase. *Science* 334, 678-683. 10.1126/science.1207056 22053050 PMC3211112

