# Supplementary material for: Loss of vacuolar-type H+-ATPase induces caspase-independent necrosis-like death of hair cells in zebrafish neuromasts
Source: Dis Model Mech. 2021 Jul 23;14(7):dmm048997. doi: 10.1242/dmm.048997 (PMC8319552; doi:10.1242/dmm.048997)
Supplement: Supplementary information [file dmm-14-048997-s1.pdf]

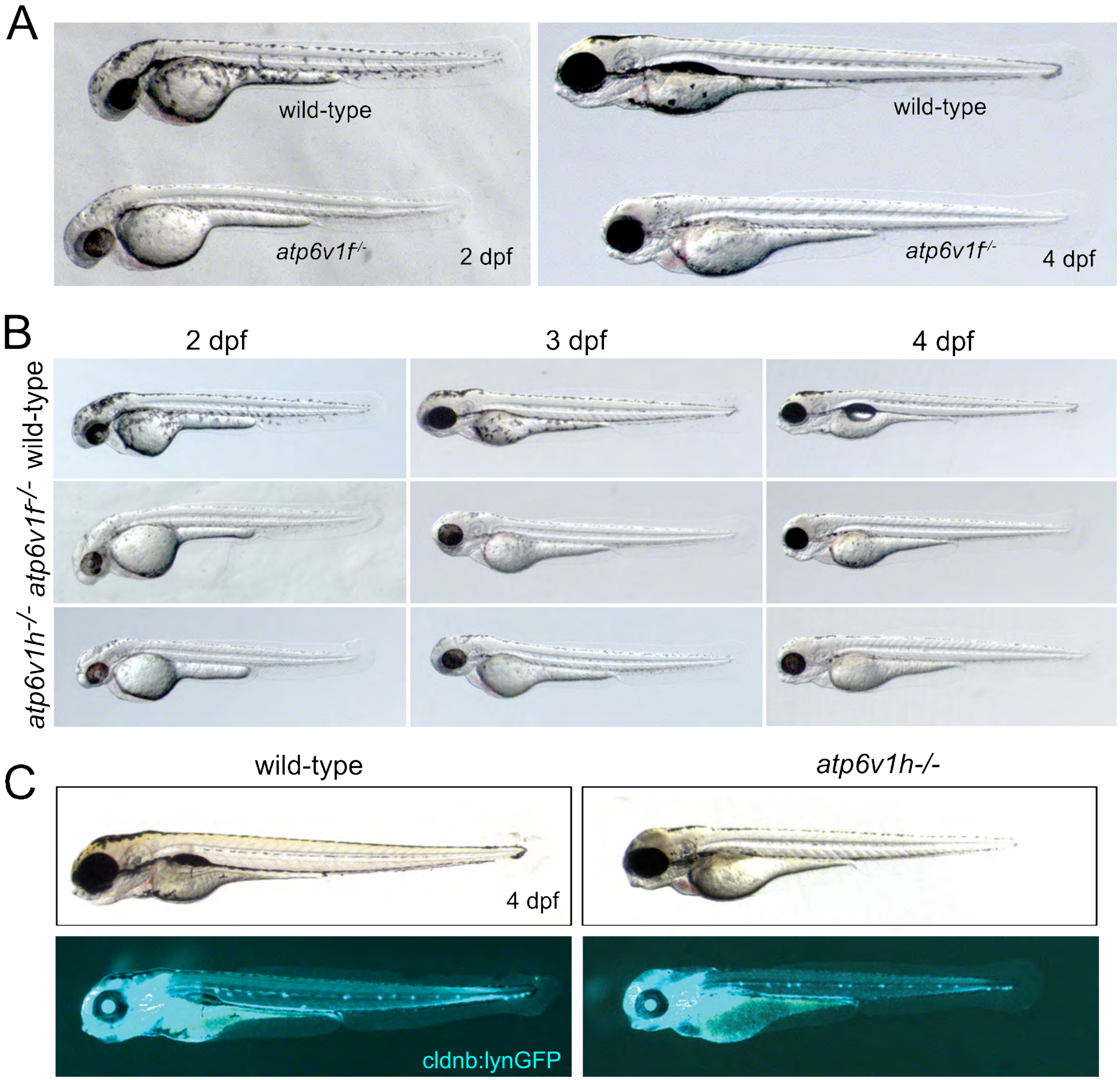

Fig. S1. **A-B.** Representative images of wild-type, *atp6v1f*$^{-/-}$ and *atp6v1h*$^{-/-}$ embryos at 2, 3 and 4 dpf. V-ATPase mutants are characterized by smaller size than wild-type, hypopigmentation, microphthalmia, facial malformations, and lack of swim bladder. **C.** Wild-type and *atp6v1h*$^{-/-}$ embryos at 4 dpf expressing the *Tg(cldnb:lynGFP)* transgene that labels lateral line neuromasts.

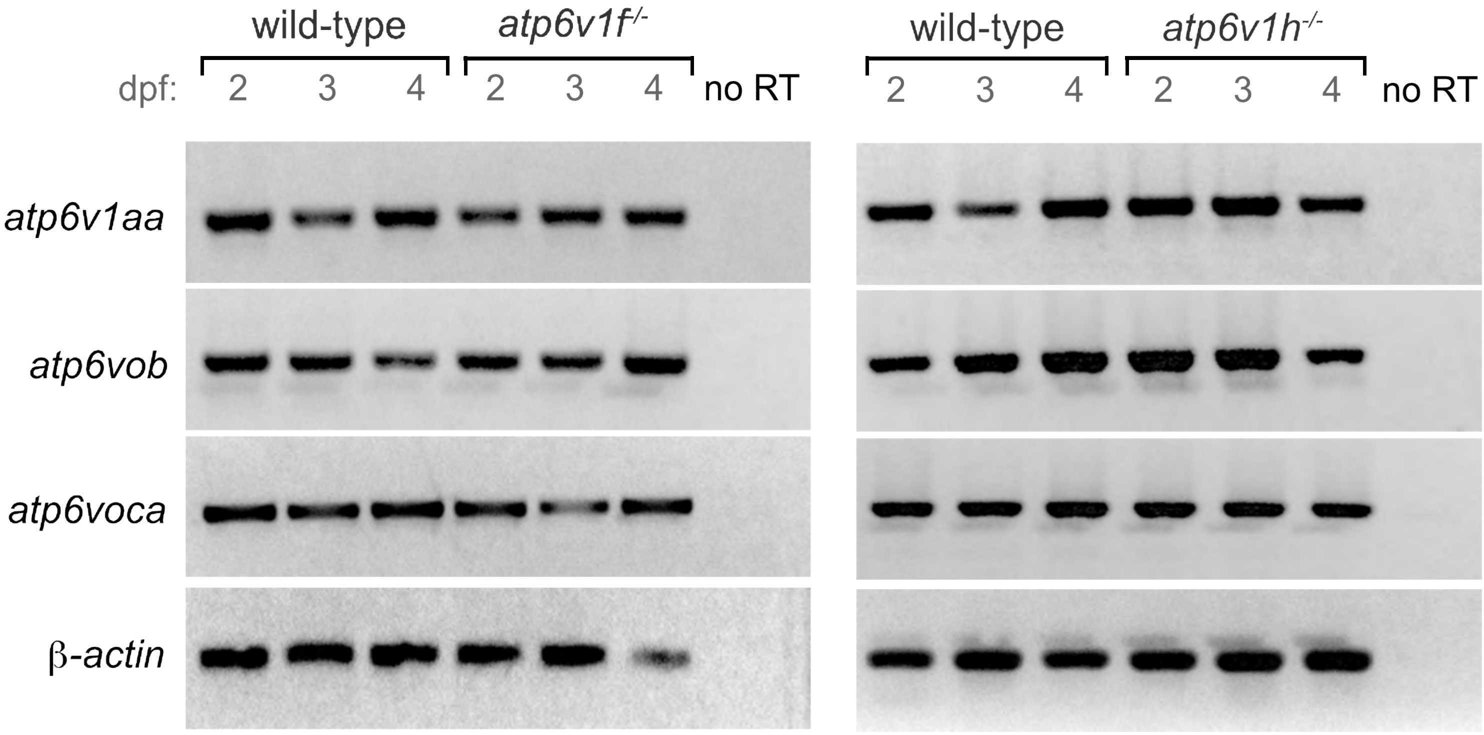

Fig. S2. Qualitative RT-PCR analysis of the representative V-ATPase subunits atp6v1aa, atp6v0b and atp6v0ca in wildtype siblings and *atp6v1f*$^{-/-}$ and *atp6v1h*$^{-/-}$ mutants at 2, 3 and 4 dpf. mRNA was extracted from whole embryo lysates. Amplification of β-*actin* was used as a positive control, and reactions lacking reverse transcriptase (no RT) served as negative controls.

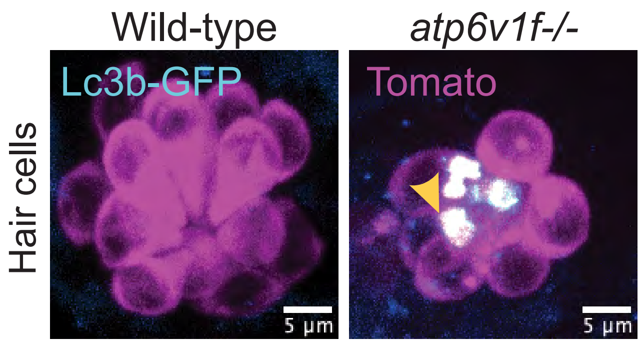

Fig. S3. Live images of wild-type and *atp6v1f*$^{-/-}$ embryos expressing the *Tg(myo6b:tdtomato)* and *Tg(CMV:EGFP-map1lc3b)* transgenes that label hair cells (purple) and autophagosomes (white). Lc3b-GFP was found to accumulate in *atp6v1f*$^{-/-}$ hair cells.

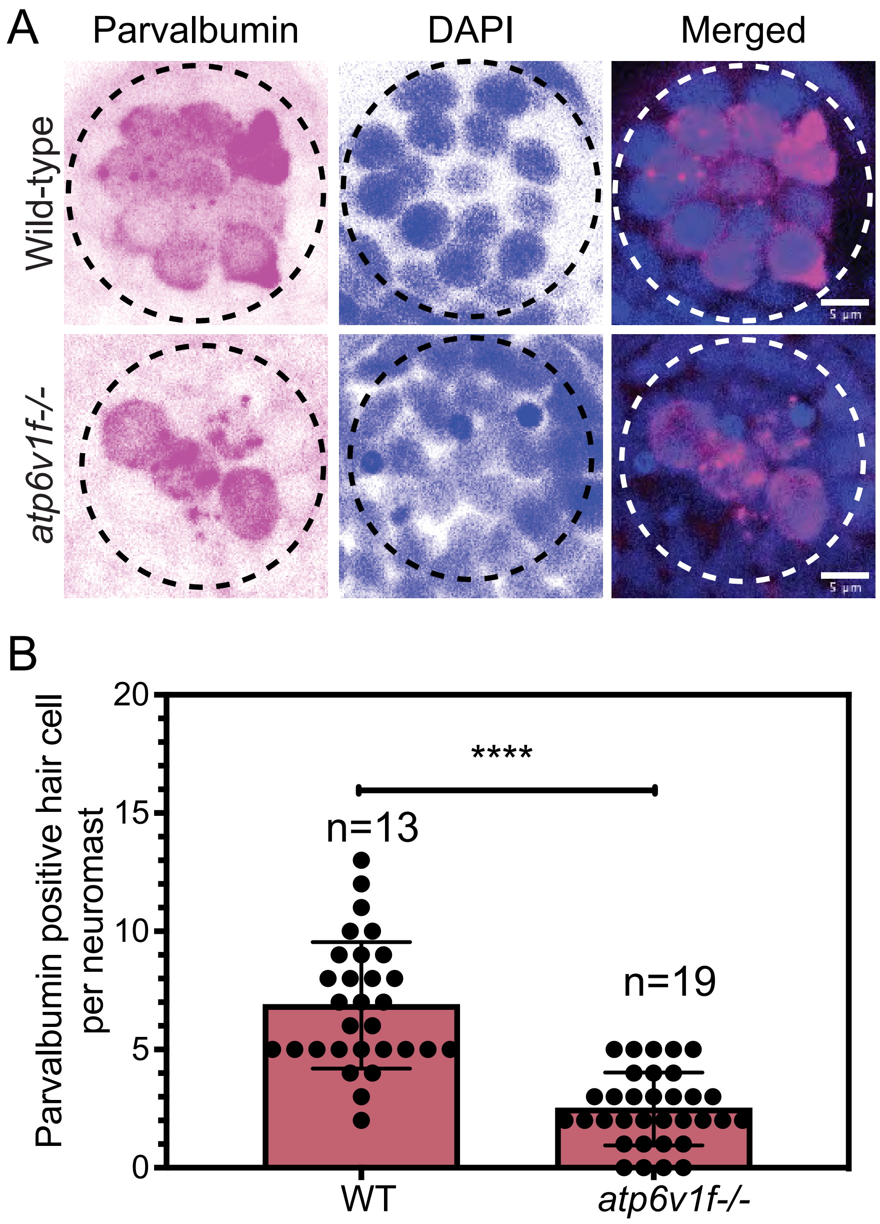

Fig. S4. **A.** Parvalbumin immunostaining labels hair cells (magenta) in wild-type and *atp6v1f*$^{-/-}$ neuromasts. DAPI stains nuclei (blue). **B.** Quantification of parvalbumin positive hair cell per neuromast in wild-type WT and *atp6v1f*$^{-/-}$. The number of parvalbumin positive hair cells is reduced in *atp6v1f*$^{-/-}$ mutant neuromasts. Each data point represents an individual neuromast measured in n=number of embryos. ****$p<0.0001$ by unpaired t-test with Welch's correction.

Disease Models & Mechanisms • Supplementary information

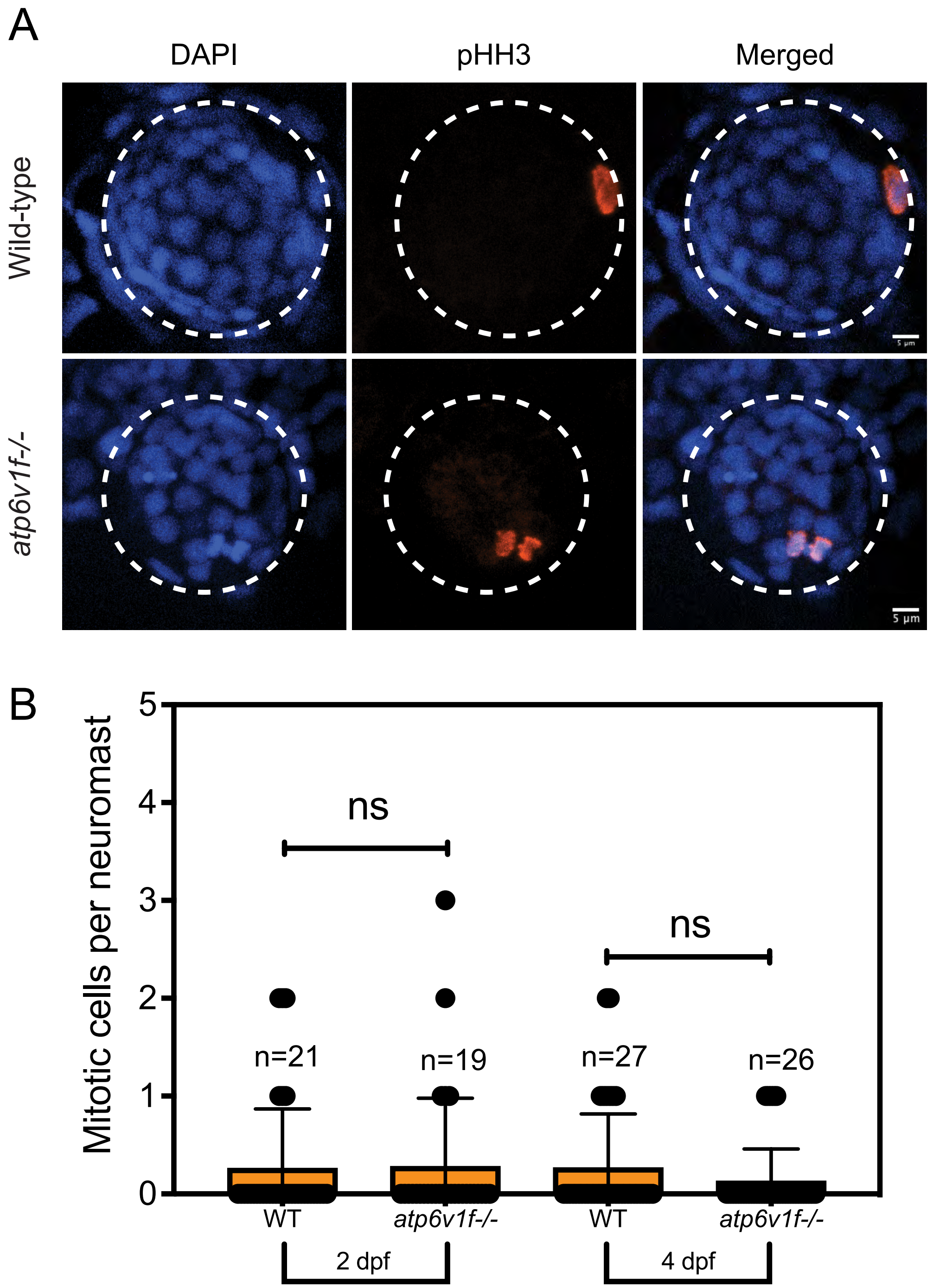

Fig. S5. A. Representative images of phospho-histone H3 (pHH3) positive cells (orange) in wild-type and *atp6v1f*$^{-/-}$ neuromasts. DAPI stains nuclei (blue). **B.** Quantification of pHH3-positive cells in the neuromasts at both 2 and 4 dpf. n=number of embryos. ns=not significant by unpaired t-test with Welch's correction.

Disease Models & Mechanisms • Supplementary information

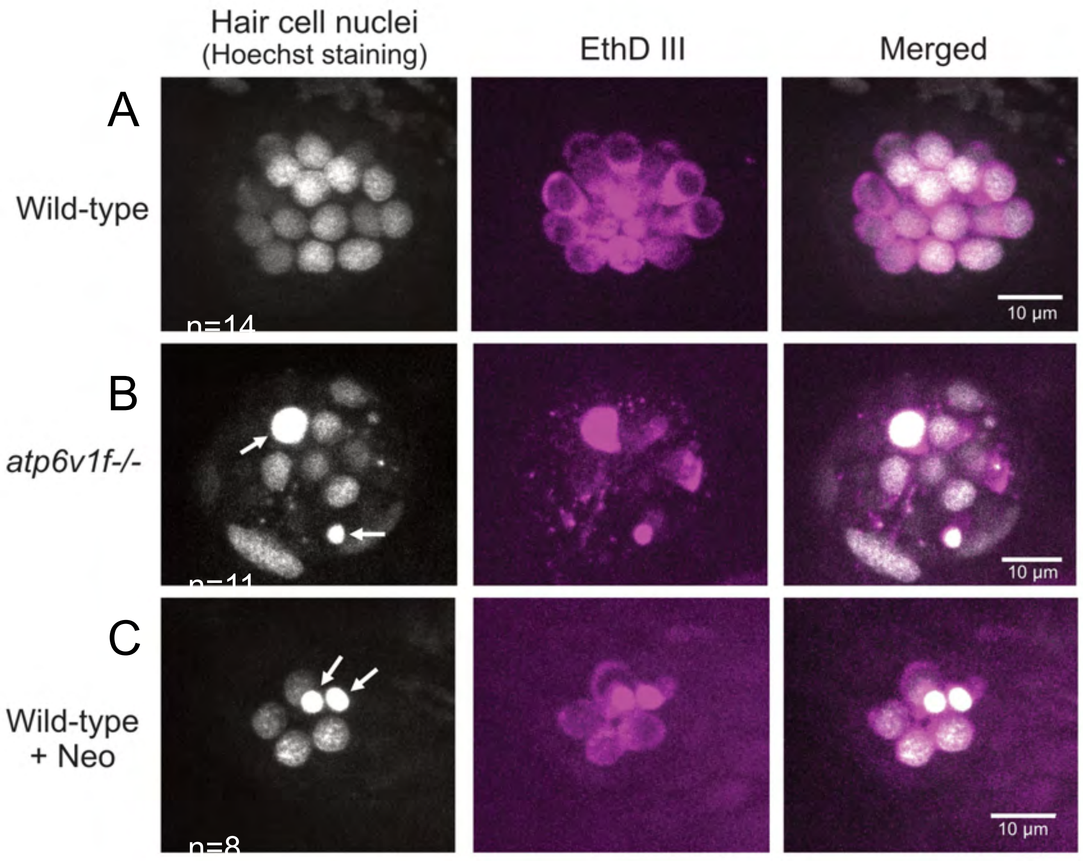

Fig. S6. A-B. 4 day old wild-type or *atp6v1f*$^{-/-}$ embryos were incubated with EthD III and Hoechst DNA stain for 30 min, and then neuromasts were imaged. Only hair cell nuclei stained with Hoechst in neuromasts. EthD III staining was found to accumulate in the cytoplasm of wild-type hair cells (A) and co-localize with Hoechst staining of pyknotic nuclei in *atp6v1f*$^{-/-}$ mutants (B). (C) As a positive control experiment, wild-type embryos were treated with 200 μM neomycin to induce hair cell death. EthD III staining co-localized with Hoechst staining of pyknotic nuclei in dying hair cells. Arrows point out pyknotic nuclei. n=number of neuromasts analyzed.

Disease Models & Mechanisms • Supplementary information

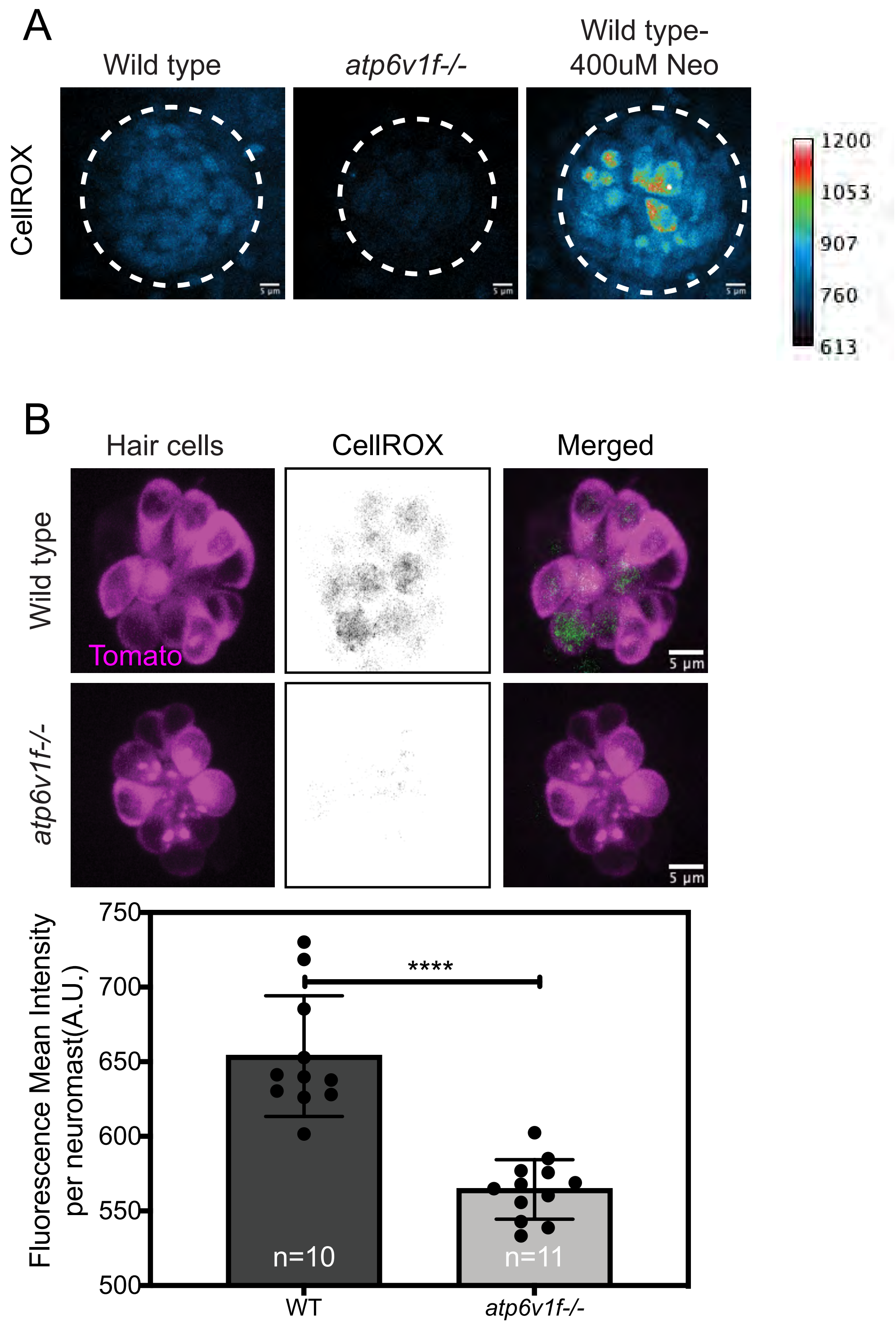

Fig. S7. A. The vital dye CellROX was used to visualize ROS at 4 dpf in live embryos. A heatmap representation of fluorescence intensity indicates CellROX staining is reduced in *atp6v1f*$^{-/-}$ neuromasts relative to wild-type siblings. As a positive control, wild-type embryos were treated with 400μM neomycin (Neo), which is known to increase ROS in hair cells. **B.** CellROX staining is reduced in *atp6v1f*$^{-/-}$ hair cells, which are marked by *Tg(myo6b:tdtomato)* transgene expression. **C.** Quantification of CellROX staining in neuromasts at 4 dpf. n= number of embryos. ****p<0.0001 by unpaired t-test with Welch's correction.

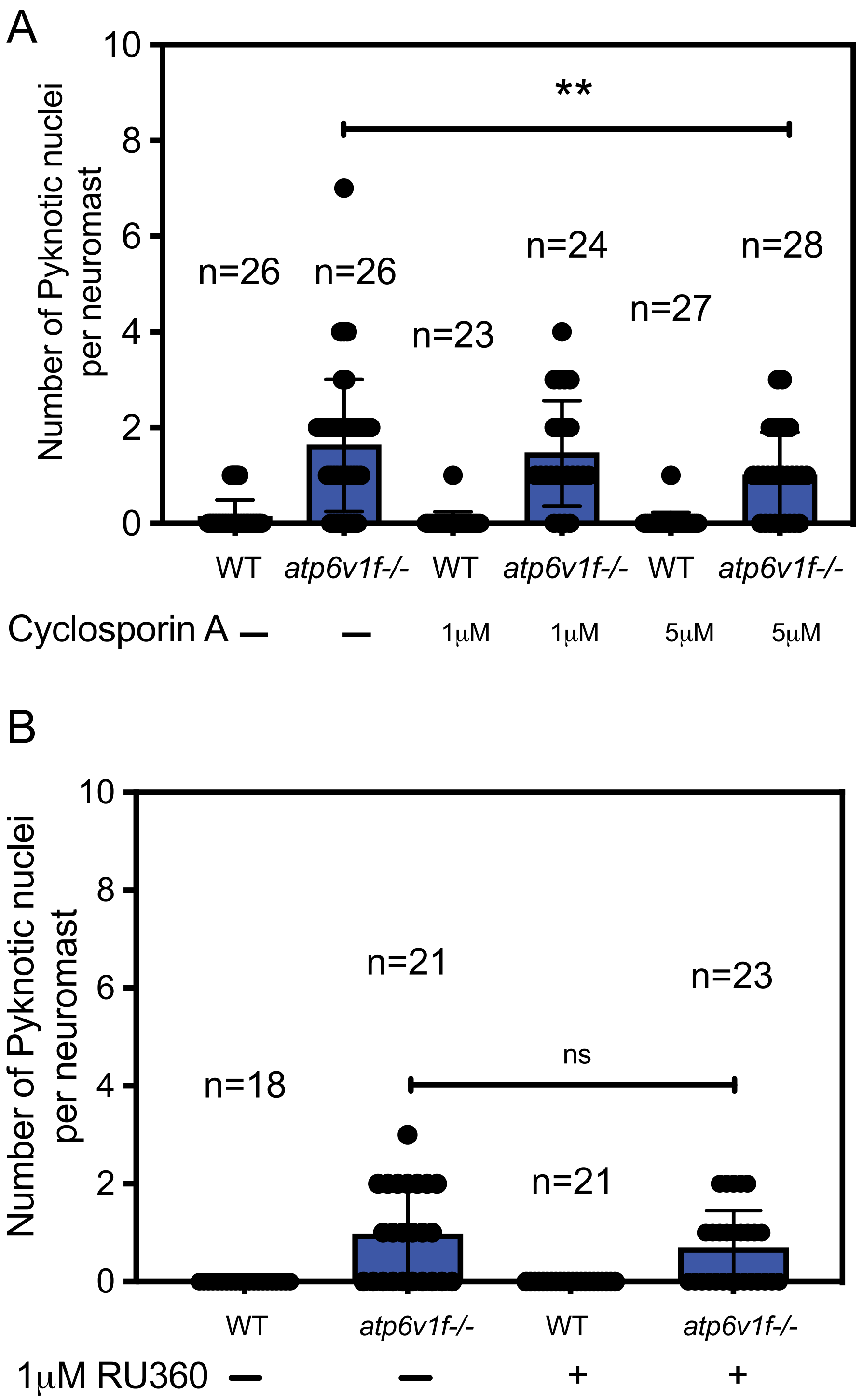

Fig. S8. A-B. The number of pyknotic nuclei per neuromast in wild-type and *atp6v1f*$^{-/-}$ embryos treated with or without CsA (A) or RU360 (B) from 2 dpf to 4 dpf. n=number of embryos. **p=0.0096 by Two-way ANOVA with Bonnferroni- Šidák post hoc test.

Disease Models & Mechanisms • Supplementary information

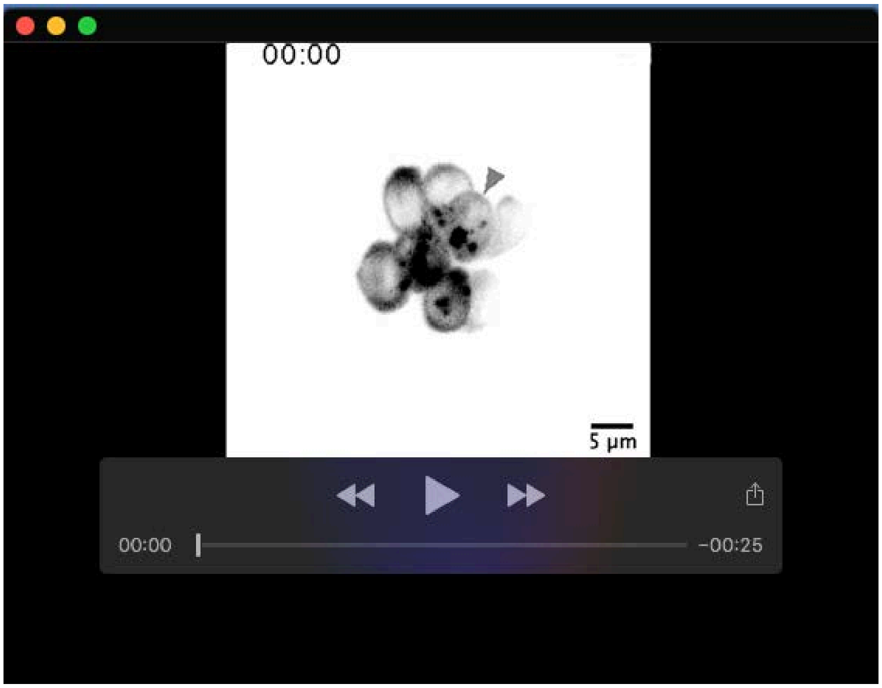

Movie 1. Live imaging of *atp6v1f*$^{-/-}$ mutant neuromast hair cells expressing *Tg(myo6b:tdtomato)* at 4 dpf. Arrowhead follows a single hair cell that swells over time. Note that fluorescent tdTomato protein accumulates into aggregates that are reminiscent of LC3b-GFP aggregates that mark autophagosomes (see Fig. 3). Time stamp=hours:minutes

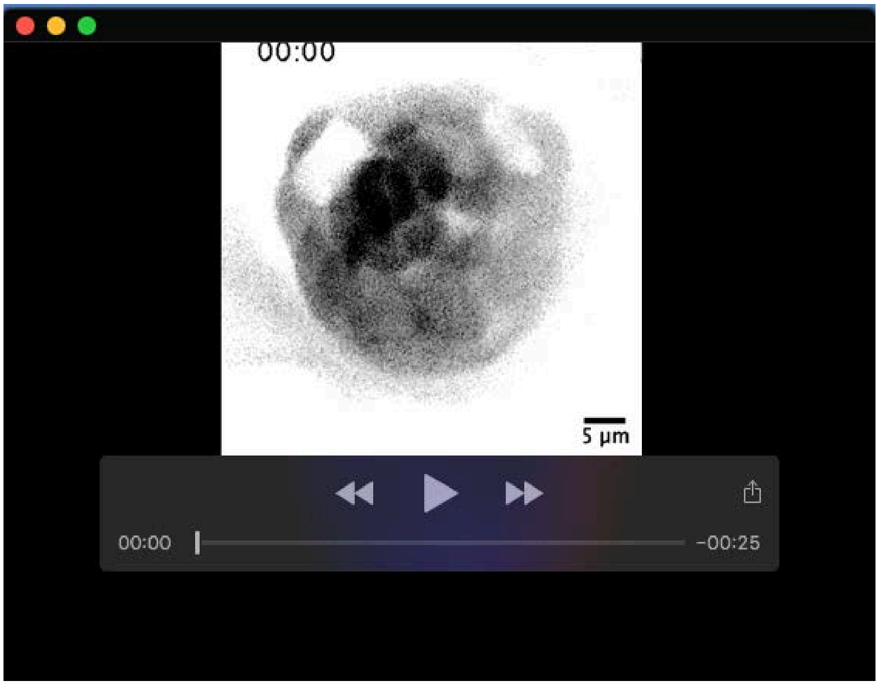

Movie 2. Live imaging of *atp6v1f*$^{-/-}$ mutant neuromast cells expressing *Tg(scm1:GFP)* at 4 dpf. Time stamp=hours:minutes

Disease Models & Mechanisms • Supplementary information

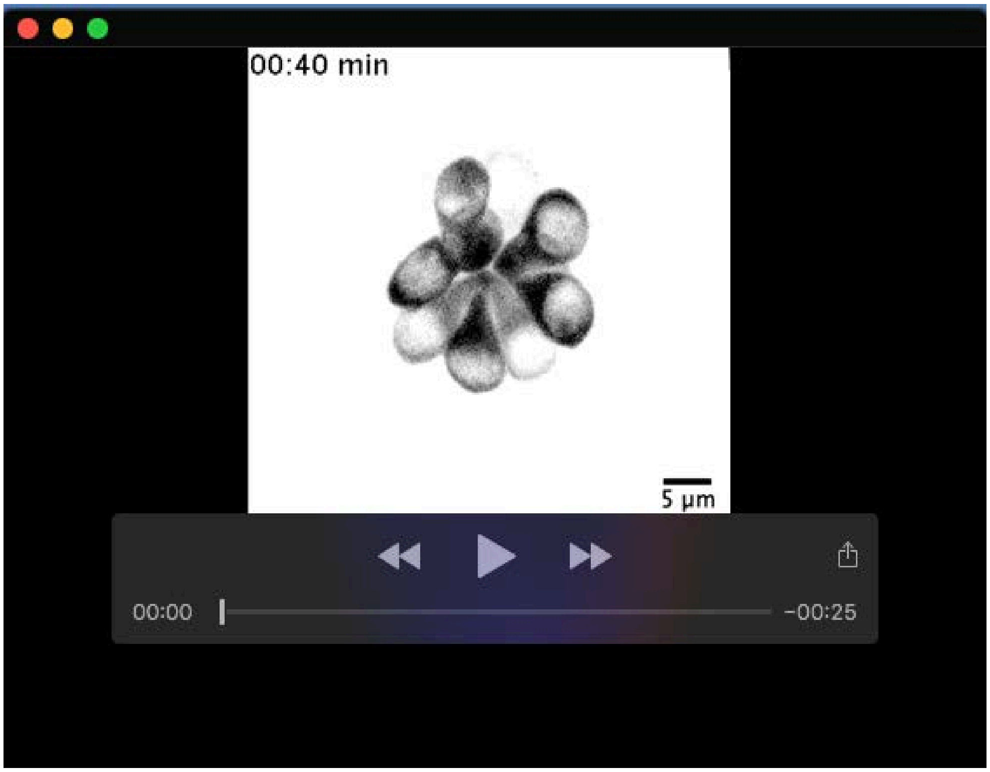

Movie 3. Live imaging of wild-type neuromast hair cells expressing *Tg(myo6b:tdtomato)* at 4 dpf. Time stamp=hours:minutes

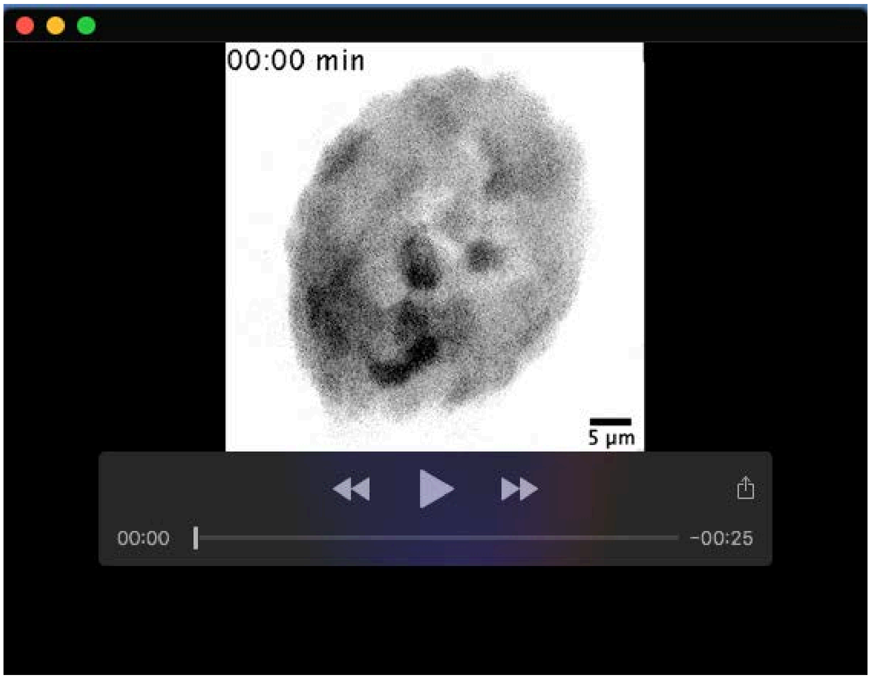

Movie 4. Live imaging of wild-type neuromast support cells expressing *Tg(scm1:GFP)* at 4 dpf. Time stamp=hours:minutes

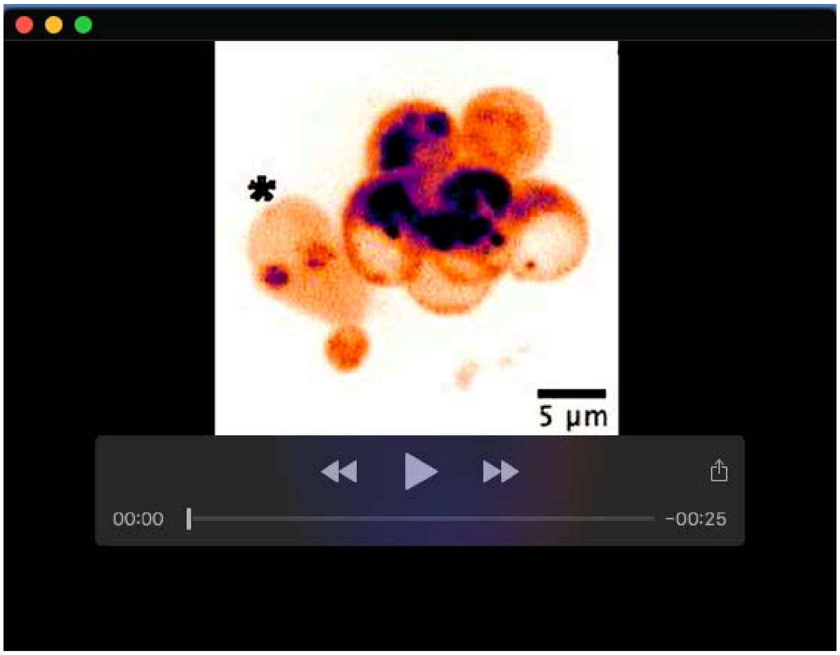

Movie 5. Live imaging of *atp6v1f*$^{-/-}$ mutant neuromast hair cells expressing *Tg(myo6b:tdtomato)* at 4 dpf. The hair cell marked by asterisk detaches from the hair cell cluster, then swells and bursts. Note that fluorescent tdTomato protein accumulates into aggregates that are reminiscent of LC3b-GFP aggregates that mark autophagosomes (see Fig. 3).

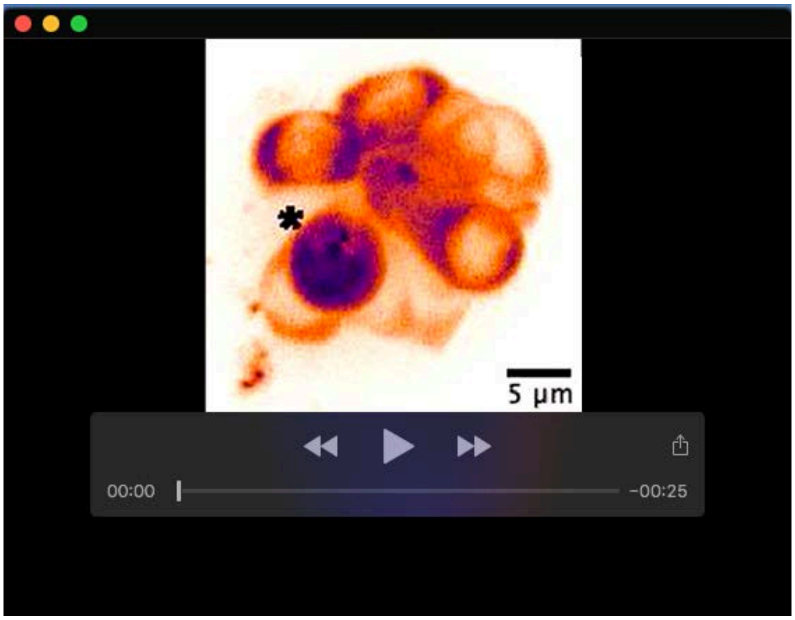

Movie 6. Live imaging of wild-type neuromast hair cells expressing *Tg(myo6b:tdtomato)* at 4 dpf treated with 10 µM $CuSO_4$ for 20 minutes at 28°C and then immediately imaged. The asterisk follows a hair cell undergoing $CuSO_4$ induced necrosis, where the hair cell disengages from the hair cell cluster, swells, and bursts.
